# Enhancing cartilage regeneration and repair through bioactive and biomechanical modification of 3D acellular dermal matrix

**DOI:** 10.1093/rb/rbae010

**Published:** 2024-02-05

**Authors:** Wei Gao, Tan Cheng, Zhengya Tang, Wenqiang Zhang, Yong Xu, Min Han, Guangdong Zhou, Chunsheng Tao, Ning Xu, Huitang Xia, Weijie Sun

**Affiliations:** Qingdao Medical College of Qingdao University, Qingdao, 266071, China; Department of Cardiothoracic Surgery, Shanghai Children’s Hospital, Shanghai Jiao Tong University School of Medicine, Shanghai, 200040, China; Department of Plastic surgery, Shanghai Key Laboratory of Tissue Engineering, Shanghai Ninth People’s Hospital, Shanghai Jiao Tong University School of Medicine, Shanghai, 200023, China; Department of Orthopaedics, The First Affiliated Hospital of Shandong First Medical University, Jinan, 266299, China; Department of Thoracic Surgery, Shanghai Pulmonary Hospital, School of Medicine, Tongji University, Shanghai, 200433, China; Department of Orthopedic Surgery, Shanghai Eighth People's Hospital, Shanghai, 200235, China; Department of Plastic surgery, Shanghai Key Laboratory of Tissue Engineering, Shanghai Ninth People’s Hospital, Shanghai Jiao Tong University School of Medicine, Shanghai, 200023, China; Department of Orthopaedics, Ninety-seventh Hospital of the Chinese People's Liberation Army Navy, Qingdao, 266071, China; Department of Orthopaedic Surgery, Shanghai Sixth People's Hospital Affiliated to Shanghai Jiao Tong University School of Medicine, Shanghai, 200025, China; Department of Orthopedic Surgery, Shanghai Eighth People's Hospital, Shanghai, 200235, China; Department of Plastic Surgery & Jinan Clinical Research Center for Tissue Engineering Skin Regeneration and Wound Repair, The First Affiliated Hospital of Shandong First Medical University, Jinan, 266299, China; Department of Infectious Diseases, The First Affiliated Hospital of Anhui Medical University, Shushan, Hefei, 230022, China

**Keywords:** acellular dermal matrix, three-dimensional scaffolds, cartilage regeneration, small intestinal submucosa, calcium sulfate hemihydrate

## Abstract

Acellular dermal matrix (ADM) shows promise for cartilage regeneration and repair. However, an effective decellularization technique that removes cellular components while preserving the extracellular matrix, the transformation of 2D-ADM into a suitable 3D scaffold with porosity and the enhancement of bioactive and biomechanical properties in the 3D-ADM scaffold are yet to be fully addressed. In this study, we present an innovative decellularization method involving 0.125% trypsin and 0.5% SDS and a 1% Triton X-100 solution for preparing ADM and converting 2D-ADM into 3D-ADM scaffolds. These scaffolds exhibit favorable physicochemical properties, exceptional biocompatibility and significant potential for driving cartilage regeneration *in vitro* and *in vivo*. To further enhance the cartilage regeneration potential of 3D-ADM scaffolds, we incorporated porcine-derived small intestinal submucosa (SIS) for bioactivity and calcium sulfate hemihydrate (CSH) for biomechanical reinforcement. The resulting 3D-ADM+SIS scaffolds displayed heightened biological activity, while the 3D-ADM+CSH scaffolds notably bolstered biomechanical strength. Both scaffold types showed promise for cartilage regeneration and repair *in vitro* and *in vivo*, with considerable improvements observed in repairing cartilage defects within a rabbit articular cartilage model. In summary, this research introduces a versatile 3D-ADM scaffold with customizable bioactive and biomechanical properties, poised to revolutionize the field of cartilage regeneration.

## Graphical abstract

**Figure:**
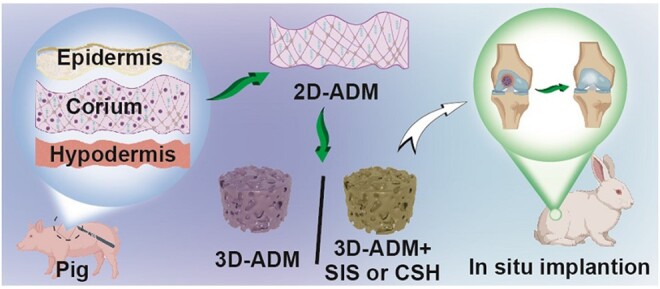

## Introduction

Articular cartilage injury (ACI) refers to the damage or destruction of the cartilage on the surface of joints. There are various factors that can lead to ACI, including accidental falls, sports injuries or accidents resulting in joint twists or impacts; prolonged or excessive use of a joint; inflammatory joint diseases, such as rheumatoid arthritis; and other inflammatory conditions [1]. Repairing articular cartilage damage is challenging due to its limited self-repair ability caused by the absence of blood vessels and nerves [2]. Currently, the clinical treatment methods for ACI can provide some relief and functional improvement, but they cannot fully repair the damaged cartilage tissue and have certain risks and limitations. For instance, conservative treatments, such as the use of painkillers, non-steroidal anti-inflammatory drugs and physical therapy, typically only alleviate pain and inflammation and cannot genuinely repair the damaged cartilage tissue. Moreover, conservative treatment often yields limited effectiveness and requires a lengthy recovery period for severe joint cartilage injuries [3]. Surgical interventions, including arthroscopic surgery, cartilage transplantation and joint replacement, also have their drawbacks. Arthroscopic surgery is a minimally invasive procedure; however, it still carries surgical risks, such as infection, bleeding and nerve damage. Additionally, the efficacy of arthroscopic surgery may be limited for severe joint cartilage injuries, as it cannot restore the original structure of the damaged cartilage. Cartilage transplantation is constrained by the limited availability of donor cartilage and the risk of complications, such as immune rejection. Joint replacement surgery, which is a more invasive procedure, necessitates a lengthy recovery period. Furthermore, joint replacement surgery is typically reserved for severe joint degeneration and damage and is not suitable for all patients [4]. Tissue engineering technology offers a new avenue for cartilage injury repair, particularly through the utilization of biofunctional scaffolds with suitable 3D porous structures [5]. Natural materials, despite the availability of synthetic alternatives, exhibit superior attributes, such as strong biocompatibility, heightened biofunctional activity and an environment mimicking native conditions [6]. These characteristics influence cell morphology, phenotype, movement, and ultimately support cell growth, proliferation and differentiation [7–9]. However, creating a natural material with an optimal structure and function for cartilage regeneration remains a challenge [10].

Decellularized extracellular matrix (DEM), a critical component of natural materials, has garnered attention for its potential in cartilage tissue engineering. DEM preserves the structure and functional proteins of the extracellular matrix (ECM) while removing foreign cell antigens [11]. Acellular cartilage matrix (ACM) was initially promising as a natural microenvironment for chondrogenesis, but its use is constrained by limited access to homogeneous cartilage sources and donor-related concerns [12]. While heterogeneous cartilage sources are plentiful, their dense structures necessitate complex processes for cell removal, often resulting in structural damage to the ECM ultrastructure, loss of growth factors and lingering chemical reagents [13]. In this context, decellularized matrices derived from other tissues, such as umbilical cord, dermis, adipose tissue and tendon have gained traction for cartilage repair [14]. Among these, acellular dermal matrix (ADM) stands out due to its wide availability, cost-effectiveness, ease of procurement and relatively loose structure, which enables the preservation of ECM ultrastructure and nutritional factors post-decellularization [15]. Our recent study reveals that ADM scaffolds outperform ACM, displaying heightened cell adhesion rates and stronger cartilage regeneration potential [16]. Therefore, the utilization of ADM for cartilage injury repair holds significant promise.

Given the distinct structural and functional requirements of cartilage tissue, scaffolds for cartilage regeneration must possess a flexible 3D shape, porous structure conducive to cell adhesion and proliferation and appropriate biomechanical strength for shape retention [17]. Yet, native ADM is inherently 2D, posing a challenge in creating a suitable 3D porous scaffold for cartilage regeneration [18]. Although researchers have explored converting 2D-ADM scaffolds into 3D-ADM scaffolds for cartilage repair, outcomes have been unsatisfactory [19]. This is due to factors, such as vulnerability of the dermis to damage during decellularization, difficulties in fabricating 3D ADM scaffolds and the need for enhanced biological activity and biomechanical properties to support cartilage regeneration [20]. Thus, finding a method to transform 2D-ADM scaffolds into 3D structures while preserving ADM’s ultrastructure and nutritional factors is crucial. Furthermore, in order to achieve the repair of cartilage, biocompatibility and affinity toward modified materials or bioactive factors are the core requirements of ADM scaffolds. Firstly, during the process of articular cartilage regeneration, ADM scaffolds need to interact favorably with surrounding tissues and cells to ensure cell adhesion, proliferation and differentiation. The material and surface characteristics of ADM scaffolds should be recognized and accepted by cells, without causing adverse reactions or rejection. Therefore, ADM scaffolds must possess good biocompatibility. Moreover, during articular cartilage regeneration, the introduction of bioactive factors (such as growth factors, cytokines and ECM) or other modified materials onto the surface or within the ADM scaffold can effectively regulate cell growth, differentiation and matrix synthesis. As a result, ADM scaffolds need to exhibit affinity toward these modified materials or bioactive factors to achieve the desired biological functions. Thus, by possessing biocompatibility and affinity toward modified materials or bioactive factors, ADM scaffolds can provide good cell adhesion and release of bioactive factors, promoting the proliferation and differentiation of chondrocytes, as well as the synthesis of cartilage matrix [21].

Decellularization methods encompass physical, chemical and enzymatic approaches. While physical treatments like low-temperature freeze-thawing dissolve tissue cells effectively, they necessitate complex subsequent steps for membrane and cellular content removal [22]. Chemical methods, like sodium hydroxide, eradicate growth factors but can diminish ECM mechanical properties [23]. Low concentrations of sodium dodecyl sulfate (SDS) and Triton X-100 can remove cell nuclei while retaining tissue mechanical structures [24]. Enzymatic approaches, such as trypsin, selectively eliminate undesirable components. Initially detrimental to elastin and collagen, low-concentration trypsin has been found to spare collagen matrix microstructure in enzymatically decellularized ADM [25]. Consequently, low-concentration trypsin becomes a viable option for cell removal.

Building on this analysis, our study employed 0.125% trypsin along with 0.5% SDS and 1% Triton X-100 for decellularization of 2D-ADM. Subsequently, freeze-drying and 1-ethyl-3-(3-dimethylaminopropyl) carbodiimide hydrochloride (EDC) cross-linking transformed 2D-ADM scaffolds into 3D counterparts. Chondrocytes were seeded onto these 3D-ADM scaffolds, followed by evaluation of biocompatibility, *in vitro* and *in vivo* cartilage regeneration capacities.

It is worth noting that in order to address the potential limitations of bioactivity and biomechanical properties in pure 3D-ADM scaffolds, appropriate modifications should be introduced. small intestinal submucosa (SIS) is commonly used in cartilage tissue engineering to enhance the bioactivity of scaffolds, due to the following reasons: (i) the SIS is rich in various bioactive substances, such as growth factors, cytokines and collagen, among others. These substances can be released from the scaffold, promoting cell proliferation and differentiation, thus facilitating cartilage tissue generation and regeneration [26]. (ii) SIS possesses a native cell-adhesive structure, facilitating cell attachment and growth. Cell attachment and growth are critical for cartilage repair, and the support and bioactive factors provided by the submucosa promote favorable cell adhesion and growth on the scaffold. (iii) SIS exhibits good immunocompatibility and biocompatibility, thus minimizing the risk of immune rejection when implanted in the body [27]. Calcium sulfate hemihydrate (CSH) is commonly employed in cartilage tissue engineering to improve the biomechanical properties of scaffolds, owing to the following reasons: (i) the incorporation of CSH enhances the mechanical strength of the scaffold, preventing collapse or loss of shape of the cartilage tissue. (ii) CSH exhibits controllable degradation characteristics, with degradation rate *in vivo* adjusted to match the growth and repair processes of cartilage tissue [28]. (iii) CSH is a biodegradable material with good biocompatibility [29]. Therefore, to enhance bioactivity and biomechanical properties, we introduced SIS and CSH to create 3D-ADM+SIS and 3D-ADM+CSH scaffolds, respectively. These were employed to repair articular cartilage defects in a rabbit model, further assessing *in situ* cartilage repair effects.

## Materials and methods

### Preparation of 2D-ADM, 3D-ADM, 3D-ADM+SIS and 3D-ADM+CSH scaffolds

Initially, pig skin underwent tissue excision via a dermatome to procure pig dermis. Subsequently, the dermis underwent sequential immersion in a 0.125% trypsin solution (Gibco, USA), followed by a 0.5% SDS solution (Sigma, Germany) and a 1% Triton X-100 solution (Sigma, Germany) for decellularization. Subsequent treatment involved exposing the decellularized dermis to a 0.2% peracetic acid solution (OTK, China) for viral inactivation. A final step included immersion in an ether solution (Clariant, China) for defatting, followed by freeze-drying to yield the 2D-ADM.

The dried 2D-ADM was ground and crushed for 5 min to generate ADM powder. Subsequently, 0.5 g of ADM powder was mixed with 10 ml of purified water in a beaker, and the mixture was mechanically stirred to achieve homogeneity. This slurry was then molded, frozen at −15°C for 2 h, and subsequently freeze-dried for 24 h to form a pre-scaffold. This pre-scaffold underwent a 12-h cross-linking process in a centrifuge tube containing 15 ml of a 2% EDC solution (ThermoFisher, China). Following cross-linking, the scaffold was rinsed with purified water to eliminate residual cross-linking agents. A second freezing step at −15°C for 2 h was followed by an additional 24-h freeze-drying cycle, yielding the final 3D-ADM scaffold, which was stored at room temperature. For further detailed procedures, please refer to patent CN 114306755 B.

Modified 3D-ADM scaffolds, namely 3D-ADM+SIS and 3D-ADM+CSH scaffolds, were fabricated using the procedure detailed in the section ‘Preparation of 2D-ADM, 3D-ADM, 3D-ADM+SIS and 3D-ADM+CSH scaffolds’. 0.5 g ADM powder and 0.1 g porcine-derived SIS powder (Regen, China) were dissolved into 10 ml ddH_2_O to fabricate 3D-ADM+SIS scaffolds. In addition, 0.5 g ADM powder and 0.1 g CSH powder (Sigma-Aldrich, Germany) were dissolved into 10 ml ddH_2_O to fabricate 3D-ADM+CSH scaffolds.

### Evaluation of dermal tissue decellularization

Undecellularized dermal tissue (native dermis) and decellularized 2D-ADM samples were initially fixed in 4% paraformaldehyde for 24 h. Following fixation, samples underwent dehydration using a graded ethanol series automatic dehydrator (Leica, ASP300S), subsequent paraffin embedding, sectioning at a 5 μm thickness and hematoxylin and eosin (H&E) staining using routine methods.

Both undecellularized and decellularized samples were fixed at room temperature in 4% paraformaldehyde and subjected to triple phosphate buffered saline (PBS) washes. Subsequently, a 4',6-diamidino-2-phenylindole (DAPI) (Gibco, USA) staining solution was applied to cover the cartilage sample surfaces, followed by a 5-min incubation at room temperature. After DAPI staining, the samples underwent triple 5-min PBS washes. Nuclear staining was observed utilizing a confocal microscope (Leica Thunder imager 3D, 20×, Germany).

A DNA quantification assay kit (Gibco, USA) was employed as per the manufacturer’s guidelines to quantify DNA content in both undecellularized and decellularized cartilage samples. Briefly, samples were digested in a proteinase K solution (250 μg/ml, 1 ml) at 56°C for 12 h and then centrifuged at 12 000 rpm for 10 min to collect the supernatants. Then, 28.7 μl of the standard solutions and supernatants were added to the wells of an opaque 96-well plate, followed by adding 71.3 μl of PicoGreen solutions and 100 μl of Tris−EDTA buffer. DNA content was also measured using a nucleic acid protein quantitation detector (Nanodrop 2000, Thermo Fisher, Waltham, MA). Similarly, GAG quantification kit (Gibco, USA) was used to detect GAG content in Taian dermis and 2D-ADM. Tissue samples were processed and analyzed using the hydroxyproline assay kit following the kit’s protocol. Hydroxyproline content was measured in triplicate for each group sample. The collagen content of the test samples was calculated based on the hydroxyproline content as ∼10% of the total collagen content, compared to the standard curve generated by measuring the absorbance values of the standard solutions. Fourier-transform infrared (FTIR) spectra of elastin in native dermis and decellularized 2D-ADM were obtained utilizing an FTIR spectrometer (Thermo Scientific Nicolet iS20, USA) in the range of 400–4000 cm^−1^.

### Characterization of 2D-ADM, 3D-ADM, 3D-ADM+SIS and 3D-ADM+CSH scaffolds

Initially, the overall appearance of the scaffolds was documented using a digital camera (Sony, Japan). Subsequently, both groups of scaffolds underwent vacuum gold sputtering and were subjected to microstructure analysis using a scanning electron microscope (SEM, SU4800, Hitachi, Japan) at an accelerating voltage of 10 kV. Micrographs of the scaffolds were captured, and pore sizes were quantified utilizing ImageJ software [30].

Porosity measurement was conducted through the gas-ethanol displacement method. The initial volume of anhydrous ethanol was denoted as *α*. Freeze-dried scaffolds were immersed in ethanol for complete saturation over 10 min, resulting in the total volume, denoted as *β*. After scaffold removal, the remaining ethanol volume was recorded as *γ*. Porosity was computed using the formula: (*α*−*γ*)/(*β*−*γ*)×100%.

Water absorption capacity of the scaffolds was gauged. Initial dry weights of scaffolds were recorded as *W*_α_. Sequentially, scaffolds were submerged in deionized water for 2, 4, 6 and 8 min, then reweighed as *W*_β_. Water absorption capacity was determined as: (*W*_β_−*W*_α_)/*W*_α_ × 100%.

Additionally, 3D-ADM scaffolds, 3D-ADM+SIS scaffolds and 3D-ADM+CSH scaffolds were underwent compression in a dynamic mechanical analyzer (Instron-5542, USA) at a rate of 1 mm/min until compression depth reached 70% of the initial height. The Young’s modulus was then calculated from the 0–20% strain–stress curve [31]. FTIR spectra of 3D-ADM scaffolds, 3D-ADM+SIS scaffolds and 3D-ADM+CSH scaffolds were obtained utilizing an FTIR spectrometer in the range of 400–4000 cm^−1^.

### Biocompatibility assessment of 2D-ADM, 3D-ADM, 3D-ADM+SIS and 3D-ADM+CSH scaffolds

Chondrocytes were obtained and cultured following standard methods. Briefly, chondrocytes were isolated from the ear cartilage of 3-week-old male New Zealand white rabbits. These chondrocytes were cultured in Dulbecco’s Modified Eagle Medium (DMEM) (Gibco, USA) supplemented with 10% fetal bovine serum (FBS) (Gibco, USA) and 1% penicillin-streptomycin (Gibco, USA) at 37°C with 5% CO_2_. Passage 2 chondrocytes were employed for the experiments.

To assess scaffold biocompatibility, passage 2 chondrocytes (5 × 10^5^ cells/ml) were seeded into 2D-ADM, 3D-ADM, 3D-ADM+SIS and 3D-ADM+CSH scaffolds. The cells were cultivated in DMEM supplemented with 10% FBS and 1% penicillin-streptomycin, with media changes every other day. Post 1, 4 and 7 days of *in vitro* culture, samples were evaluated using a confocal microscope (Leica Thunder imager 3D, 20×, Germany) per the Live & Dead Cells Viability Assay instructions (Invitrogen, USA) to assess chondrocyte viability within the scaffolds [32]. Briefly, prepare the staining solution by mixing calcium AM, propidium iodide and diluent in a ratio of 1 μl:1  ml. Carefully wash the samples three times with PBS and then fully immerse the samples with the prepared staining solution. Incubate the samples at 37°C in the dark for 30 min, and subsequently capture images using confocal microscopy. Live & Dead stained images underwent quantification via ImageJ software to compare cell viability and the cell layer variation between the 2D- and 3D-ADM scaffolds.

For visualization of cell diffusion within the different scaffolds, F-actin and nucleus were stained using phalloidin (Yeasen, China) and DAPI, respectively. Briefly, prepare the working solution by mixing PBS with phalloidin at a ratio of 1 ml to 5 μl. Samples collected at 1, 4 and 7 days were washed twice with 37°C PBS. They were then fixed with 4% paraformaldehyde for 10 min, washed twice with PBS for 10 min each time and permeabilized with Triton X-100 for 5 min. After washing twice with PBS for 10 min each time, the samples were incubated with 200 μl of the working solution, avoiding direct light, at room temperature for 30 min. They were then washed three times with PBS for 5 min each time and counterstained with DAPI (100 nM) for ∼40 s. After washing once with PBS, the samples were observed and imaged using laser confocal microscopy. Subsequently, laser confocal microscopy was employed for observation and imaging. Cell proliferation on Days 1, 4 and 7 was quantitatively analyzed using a Cell Counting Kit-8 (CCK-8) (Dojindo, Japan). The CCK-8 assay adhered to the manufacturer’s instructions, with absorbance measurements taken at 450 nm via a spectrophotometer.

### *In vitro* assessment of cartilage regeneration using 2D-ADM, 3D-ADM, 3D-ADM+SIS and 3D-ADM+CSH scaffolds

To prepare the chondrocyte-scaffolds constructs of 2D-ADM, 3D-ADM, 3D-ADM+SIS and 3D-ADM+CSH scaffolds, first, chondrocytes were collected and prepared as a cell suspension with a concentration of 5 × 10^8^ cells/ml. Second, 2D ADM scaffolds were prepared as circular disks with a diameter of 10 mm and a thickness of 0.8 mm, while 3D-ADM, 3D-ADM+SIS and 3D-ADM+CSH scaffolds were prepared as circular disks with a diameter of 10 mm and a thickness of 2 mm. Third, the samples were placed in a 6-well plate, and 500 μl of cell suspension was added to each sample. After incubating in a cell culture incubator for 3 h to promote cell attachment, 10 ml of chondrocyte culture medium was carefully added to each well. The medium was refreshed the next day. These chondrocyte-scaffold constructs were cultured in six-well plates for 3 weeks, after which the samples were gathered for *in vitro* evaluation of cartilage formation. The culture medium encompassed DMEM, 10 ng/ml TGF-β1 (R&D Systems Inc., USA), 40 ng/ml dexamethasone (R&D Systems Inc., USA), 100 ng/ml IGF-1 (R&D Systems Inc., USA) and other supplements detailed in prior studies [33].

Initially, gross photographs of each group’s samples were taken via a digital camera, and their thickness, volume and wet weight were quantified. Collected samples were fixed in 4% paraformaldehyde for 24 h, dehydrated through a graded ethanol series, paraffin-embedded following routine protocols and sectioned at a 5 μm thickness. Evaluation of tissue structure in cartilage rings was accomplished through H&E staining. Additionally, Safranin O, toluidine blue and immunohistochemical staining for Collagen II were conducted to assess GAG and Collagen II deposition, along with Masson’s trichrome staining to visualize collagen fibers.

For GAG content determination, the Alcian blue method was employed: samples were digested using papain (Sigma, Germany), with total GAG precipitated through guanidine hydrochloride (Sigma, Germany). The dissolved GAG precipitate’s optical density was measured at 595 nm. GAG content was subsequently calculated based on a chondroitin sulfate-4-sulfate established standard curve. Quantification of Collagen II content relied on hydroxyproline analysis. Briefly, samples were hydrolyzed in an alkaline hydrolysis solution, and quantification of free hydroxyproline was performed as previously outlined. DNA quantification was carried out using the PicoGreen dsDNA assay kit (Invitrogen, USA) in accordance with the manufacturer’s instructions.

### *In vivo* assessment of cartilage regeneration using 2D-ADM, 3D-ADM, 3D-ADM+SIS and 3D-ADM+CSH scaffolds

All animal experimentation procedures were approved by the Ethics Committee of Shanghai Pulmonary Hospital (K21-355Y). As described in section ‘*In vitro* assessment of cartilage regeneration using 2D-ADM, 3D-ADM, 3D-ADM+SIS and 3D-ADM+CSH scaffolds’, chondrocyte-scaffold constructs were prepared using the aforementioned method. These constructs were subcutaneously implanted into 20 anesthetized nude mice (4-weeks-old, *n* = 5 per group), irrespective of gender (Shanghai Jiagan Experimental Animal Raising Farm, China). After 2 and 4 weeks of implantation, samples were collected and photographed. Thickness, width and wet weight of all samples were measured. Subsequently, samples obtained from each group after 2 and 4 weeks of culture underwent compression in a dynamic mechanical analyzer (Instron-5542, USA) at a rate of 1 mm/min until compression depth reached 70% of the initial height. The Young’s modulus was then calculated from the 0–20% strain–stress curve. Histological and immunohistochemical analyses, alongside quantitative assessments of DNA, GAGs and Collagen II, were carried out as previously detailed.

### *In situ* assessment of articular cartilage repair in a rabbit model

Twelve healthy New Zealand white rabbits, aged 3 months and weighing ∼2.0–2.5 kg, were divided into four groups: untreated with no scaffolds (serve as blank), 3D-ADM, 3D-ADM+SIS and 3D-ADM+CSH groups. The rabbits were anesthetized using Zoletil^TM50^ (0.35 ml/kg), and a custom-made drill was utilized to create articular defects in the trochlear groove of the rabbit knee joint (diameter: 4 mm; depth: 4 mm). Articular repair samples were collected 8-weeks post-surgery.

Initially, knee joint samples were captured using a SLR camera, and the International Cartilage Repair Society (ICRS) macroscopic scoring system (Supplementary Table S1) was employed for assessment. Subsequently, articular defect samples underwent evaluation through a micro-CT scanner (mCT-80, Scanco Medical, Switzerland) in high-resolution scanning mode, with 3D isosurface rendering conducted to visualize morphological characteristics. After fixation in 4% paraformaldehyde, samples were decalcified in a 10% ethylenediaminetetraacetic acid dihydrate solution for 3 weeks. Paraffin sections were prepared for subsequent H&E staining to evaluate tissue structure, and toluidine blue and Safranin O/Fast Green (SO/FG) staining were performed to evaluate ECM deposition. Immunohistochemical staining was conducted to confirm Collagen II expression, indicative of a cartilage-specific phenotype. Further evaluation of knee joint repair condition utilized the O'Driscoll histological scoring system (Supplementary Table S2). Quantitative analysis of GAGs and Collagen II followed previously described methods.

### Statistical analysis

Statistical analyses were conducted using GraphPad Prism 8.0 software. Quantitative data, obtained from a minimum of three independent experiments, were presented as mean ± standard deviation. *T*-test and One-way analysis of variance was utilized to determine statistical significance among different groups. Additionally, a Tukey *post-hoc* test was conducted through multiple comparisons to ascertain which groups exhibited significant differences. Differences with *P* < 0.05 were considered statistically significant, denoted as *P < 0.05, **P < 0.01 or ***P < 0.001.

## Results

### Assessment of decellularization efficiency in the dermis

**Figure 1.**
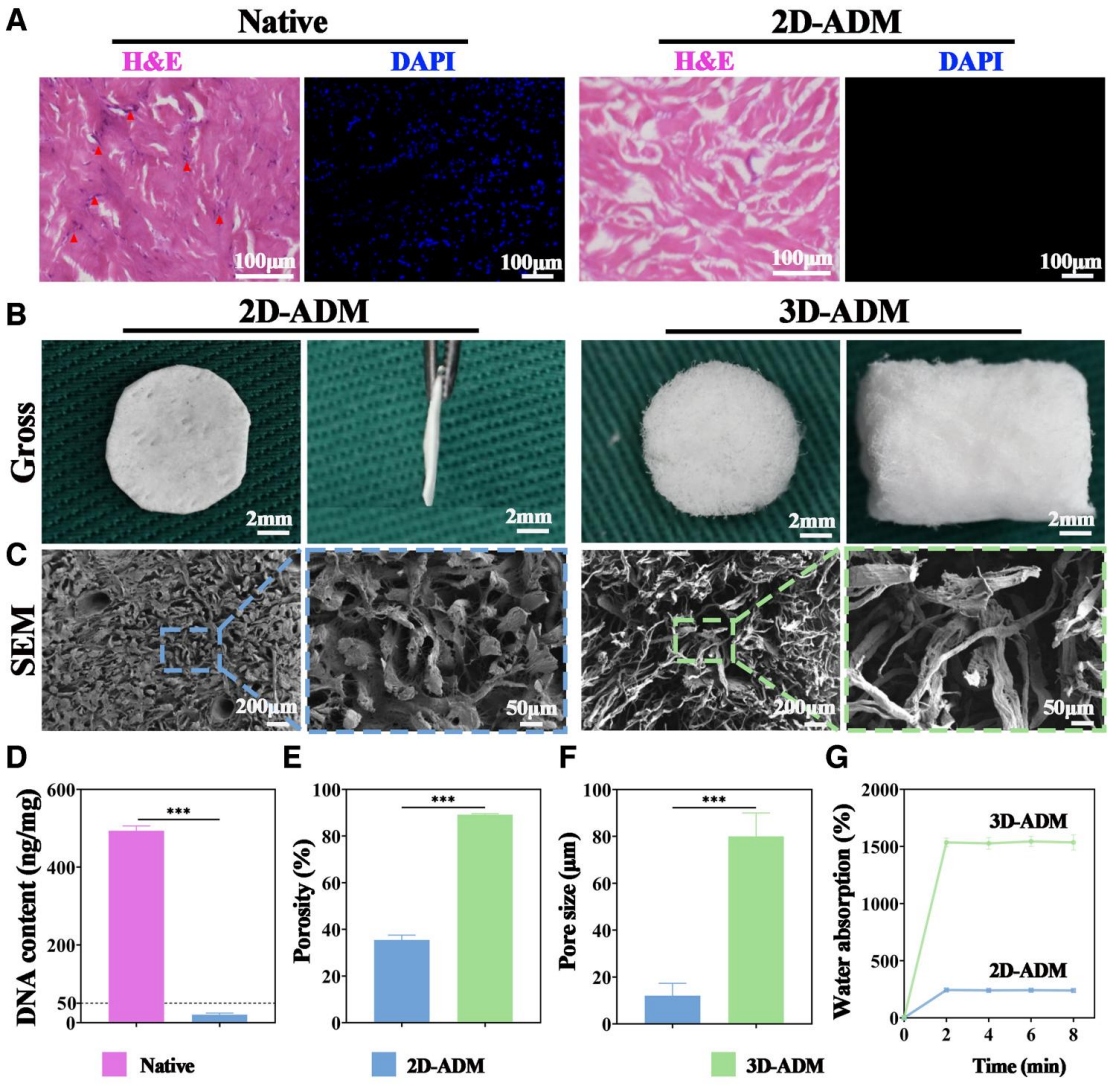
Characterization of 2D- and 3D-ADM scaffolds. (**A**) Histological staining using H&E and DAPI on natural dermis and 2D-ADM scaffolds. **(B**) Gross observation of both top and side views of 2D-ADM and 3D-ADM scaffolds. **(C**) SEM images depicting the microstructure of 2D-ADM and 3D-ADM scaffolds. The right panel provides magnified images corresponding to the left panel. **(D**) Measurement of DNA content in natural dermis and 2D-ADM scaffolds. **(E**) Evaluation of porosity in 2D- and 3D-ADM scaffolds. **(F**) Measurement of pore size in 2D- and 3D-ADM scaffolds. **(G**) Determination of water absorption capability in 2D- and 3D-ADM scaffolds. Note: ***P < 0.001.

Initially, H&E staining of non-decellularized dermis showcased deep blue chondrocyte nuclei, while in the decellularized 2D-ADM, absence of visible cell nuclei within the tissue was evident. Upon DAPI staining, native dermis displayed chondrocyte nuclei emitting blue fluorescence upon excitation, whereas no nuclei were observed in the 2D-ADM scaffolds (Figure 1A). Quantitative DNA analysis revealed a substantial reduction in DNA content within the 2D-ADM scaffolds (20.7 ng/mg), showing over 90% decrease compared to native dermis (493.3 ng/mg), resulting in a DNA content lower than 50 ng/mg (Figure 1D). These results affirm the thorough removal of cells from the dermal tissue.

In order to verify whether the decellularization process causes damage to the ECM components of natural dermis, a detection of total collagen content, GAG content and FTIR test of elastin protein was performed. The results showed that the total collagen content and GAG content showed no statistically significant difference between the natural dermis and 2D-ADM scaffolds. In addition to the characteristic peaks of amide-I and amide-II near 1650 and 1550 cm^−1^, respectively, which are common to elastin and other proteins, such as glycosaminoglycan and fibronectin, there were four characteristic absorption peaks of elastin located at 3309.8, 1651, 1539.1 and 1238.2 cm^−1^. These are associated with the N–H stretching vibration absorption peak of the amide-A band, the C = O stretching vibration absorption peak of the amide-I band, the C–N stretching vibration absorption peak of the amide-II band and the N–H bending vibration absorption peak of the amide-III band, respectively. These results indicate that elastin protein is present in both natural dermis and 2D-ADM (Supplementary Figure S1).

### Characterizations of 2D- and 3D-ADM scaffolds

**Scheme 1.**
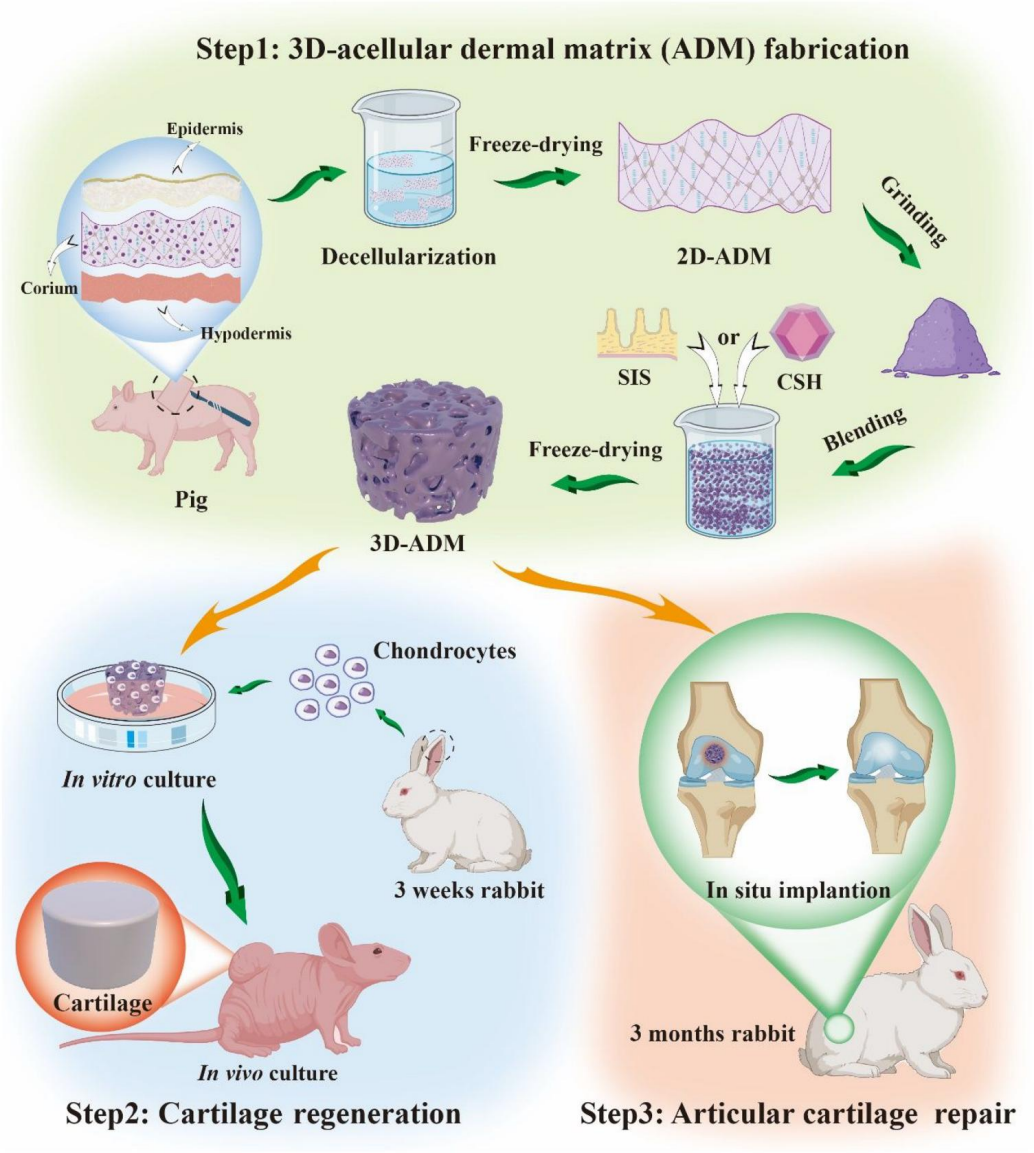
Research design. In this scheme, the research design is visually depicted in three steps: (1) the process initiated with the preparation of a 2D-ADM scaffold. A decellularization technique employing a solution comprising 0.125% trypsin and 0.5% SDS, as well as a 1% triton X-100 solution, was utilized. The ensuing phases encompassed the transformation of the 2D-ADM scaffold into both SIS and CSH modified 3D-ADM scaffolds. This transformation was achieved through a sequence of operations, including grinding, introduction of SIS or CSH, lyophilization and cross-linking. (2) Subsequent to scaffold preparation, chondrocytes were seeded onto the 3D-ADM scaffolds. These seeded scaffolds were then subjected to *in vitro* and *in vivo* assessments targeting cartilage regeneration. (3) This phase involved the utilization of the modified 3D-ADM scaffolds to repair an articular cartilage defect model in rabbits. The aim was to examine the efficacy of the modified scaffolds in facilitating cartilage repair within the *in situ* context.

Following verification of dermal decellularization, the preparation of 3D-ADM scaffolds adhered to the process outlined in Scheme 1. Macroscopic examination from the frontal perspective unveiled porcelain white appearance of the 2D-ADM scaffolds with prominent larger pores distributed on the surface, a result of hair follicle removal. In lateral view, the 2D-ADM scaffolds exhibited thinness. In contrast, the 3D-ADM scaffolds displayed uniform porousness and porcelain white appearance from the frontal view, while their lateral view indicated significantly increased thickness in comparison to 2D-ADM scaffolds (Figure 1B). SEM images further illustrated that the 2D-ADM scaffolds lacked evident pore structure, presenting only a few surface protrusions. Conversely, the 3D-ADM scaffolds showcased uniform and abundant pore structures (Figure 1C). Porosity analysis confirmed porosity exceeding 85% in 3D-ADM scaffolds, contrasting with below 40% porosity in 2D-ADM scaffolds, thereby highlighting distinct differences between the two scaffold types (Figure 1E). Pore size measurements demonstrated notably larger pores in 3D-ADM scaffolds compared to 2D-ADM scaffolds, with a difference exceeding 60 μm (Figure 1F). Mechanical performance testing revealed that the Young’s modulus of the 2D-ADM scaffolds is 846.7 kPa, surpassing that of the 3D-ADM scaffolds at 184.1 kPa (Supplementary Figure S2). Finally, water absorption analysis demonstrated superior water absorption for the 3D-ADM scaffolds at 1550%, in contrast to the less favorable water absorption of the 2D-ADM scaffolds at 245% (Figure 1G). In summation, these results underscore successful fabrication of 3D-ADM scaffolds and their superior physical attributes when compared to 2D-ADM scaffolds.

### Biocompatibility of 2D and 3D-ADM scaffolds

**Figure 2.**
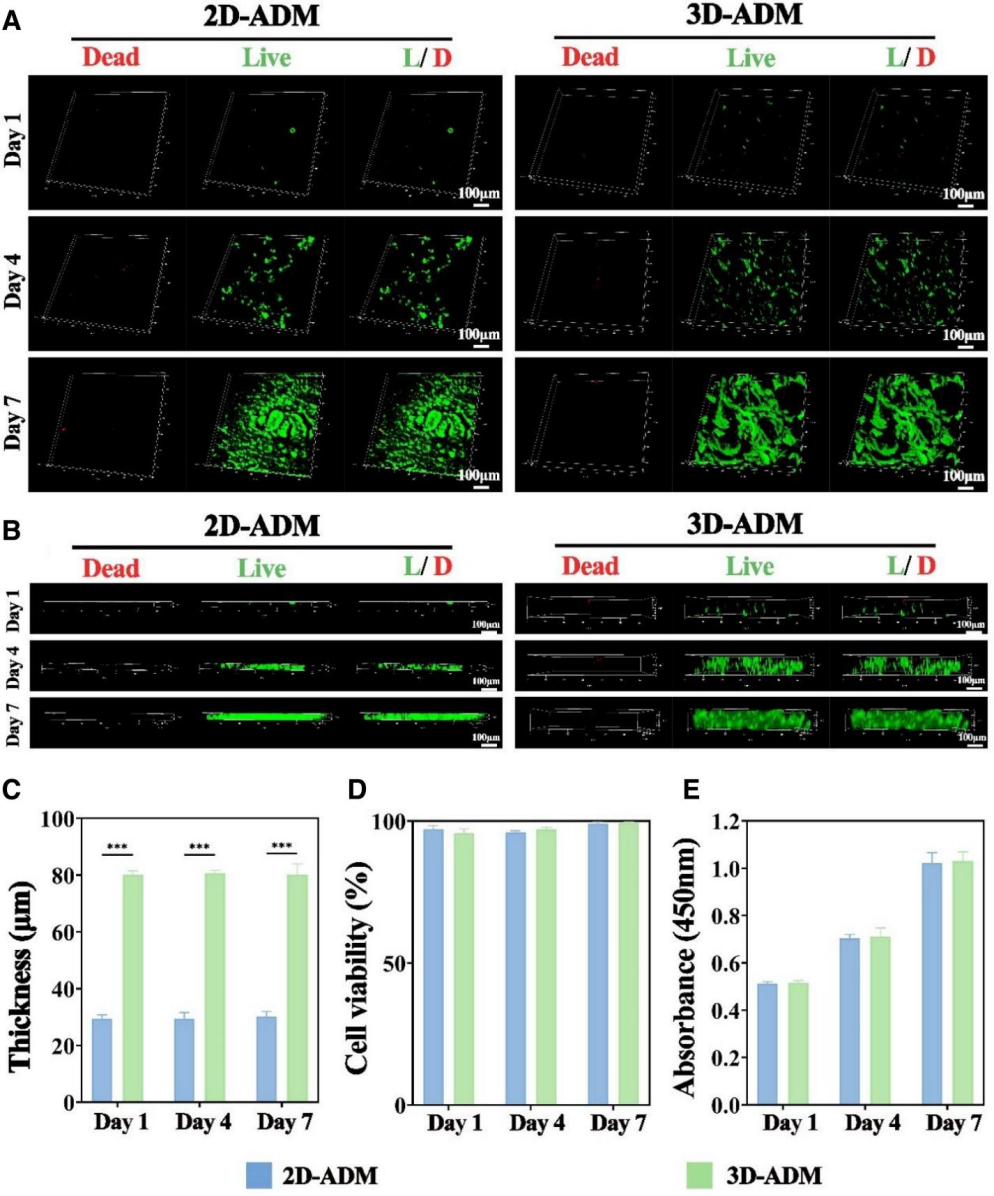
Biocompatibility of 2D- and 3D-ADM scaffolds. (**A**) Live & dead cells staining of 2D- and 3D-ADM scaffolds after 1–7 days of coculture with chondrocytes, presented from the anterior view. (**B**) Live & dead cells staining of the same scaffolds, this time viewed from the lateral perspective. (**C**) Measurement of lateral thickness for both 2D- and 3D-ADM scaffolds, obtained from the live & dead cells staining. (**D**) Assessment of cell viability in 2D- and 3D-ADM scaffolds. (**E**) Cell proliferation evaluation conducted using the CCK-8 assay on 2D- and 3D-ADM scaffolds. Note: ***P < 0.001.

Following the successful fabrication of 3D-ADM scaffolds, the assessment of biocompatibility ensued. The 3D Live & Dead Cells staining, viewed frontally, portrayed discernible cell proliferation over time in both the 2D-ADM and 3D-ADM scaffold groups, signifying absence of cytotoxicity and minimal influence on cell growth and proliferation in both scaffold variants (Figure 2A). Lateral visualization of the 3D Live & Dead Cells staining unveiled that while cell proliferation is robust in both scaffold groups, the available thickness for cell growth and proliferation in the 3D-ADM scaffold is significantly greater than that in the 2D-ADM scaffold, measuring 50 μm (Figure 2B). Quantitative analysis reinforced this finding, indicating that the thickness of the cell growth and proliferation zone in the 3D-ADM scaffolds was ∼80 μm, a significant increase from the ∼30 μm observed in the 2D-ADM scaffolds (Figure 2C). Furthermore, cell viability assays indicated no noteworthy cytotoxicity in either scaffold group (Figure 2D), and CCK-8 assay results affirmed substantial cell proliferation over the assessment period in both the 2D-ADM and 3D-ADM scaffold groups (Figure 2E). F-actin/DAPI staining illustrated satisfactory cell spreading in both scaffold groups (Supplementary Figure S3). These findings collectively establish the excellent biocompatibility of both scaffold types, with the 3D-ADM scaffolds affording greater space for enhanced cell growth and proliferation.

### *In vitro* assessment of cartilage regeneration using 2D- and 3D-ADM scaffolds

**Figure 3.**
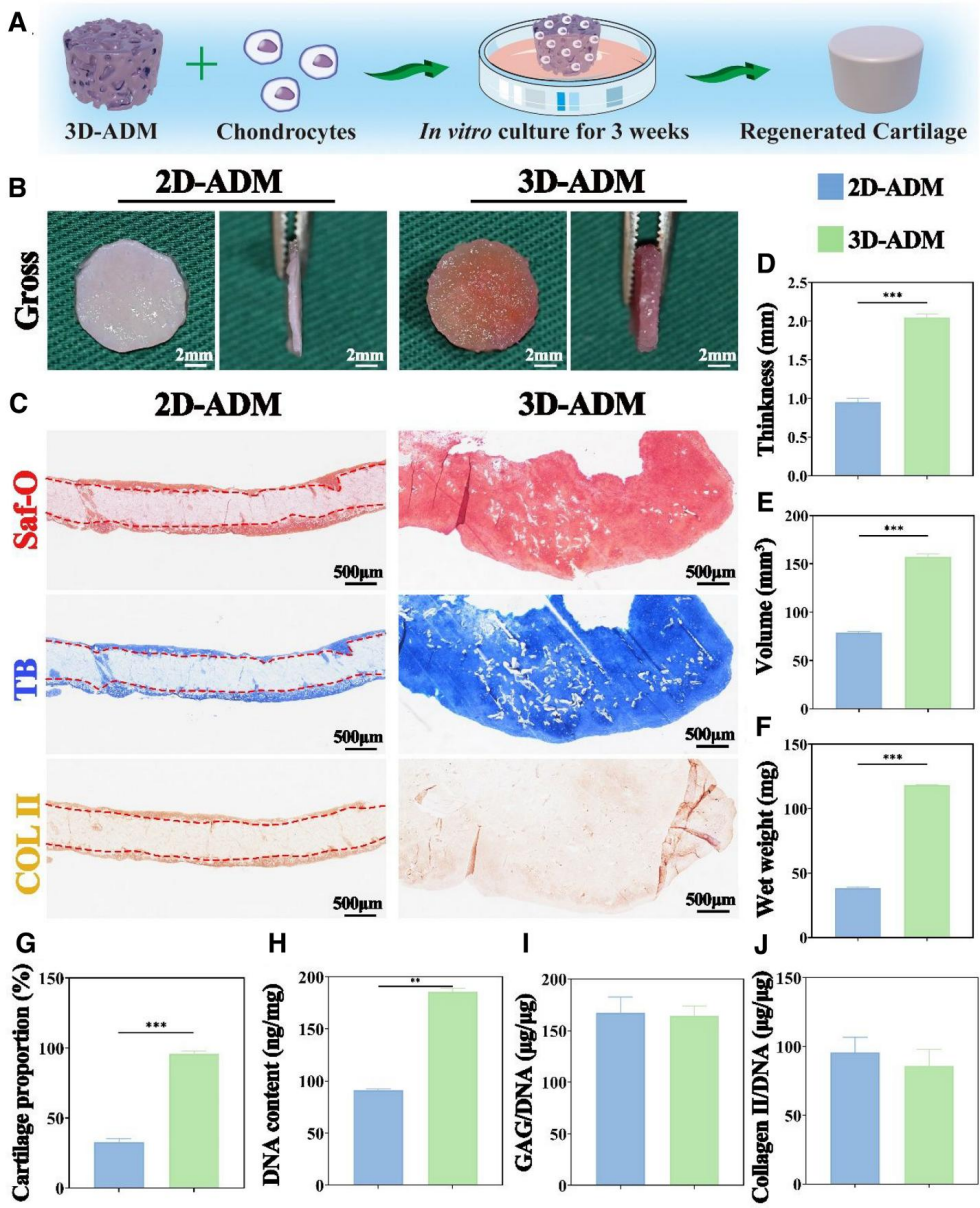
*In vitro* evaluation of cartilage regeneration using 2D- and 3D-ADM scaffolds colonized with chondrocytes for 3 weeks. (**A**) Schematic representation of the *in vitro* cartilage regeneration process employing 3D-ADM scaffolds colonized with chondrocytes. (**B**) Macroscopic observation of the regenerated cartilage, presented both from the top and side views, within 2D- and 3D-ADM scaffolds. (**C**) Staining images showcasing Saf-O, TB and immunohistochemical COL II staining of the regenerated cartilage in 2D- and 3D-ADM scaffolds. Measurement of (**D**) thickness, (**E**) volume, (**F**) wet weight, (**G**) proportion of cartilage, (**H**) DNA content, (**I**) GAG/DNA ratio and (**J**) Collagen II/DNA ratio in the regenerated cartilage. Note: **P < 0.01 and ***P < 0.001.

To evaluate the *in vitro* chondrogenic regenerative potential of the 2D-ADM and 3D-ADM scaffolds, the chondrocyte-scaffold constructs were examined after 3 weeks of culture (Figure 3A). Macroscopically, the 2D-ADM scaffolds displayed a pale pink hue with a layer of translucent white material on the surface, while the 3D-ADM scaffolds exhibited a pink color with yellow reflective components (Figure 3B). Longitudinal sections subjected to Saf-O, TB and immunohistochemical COL II staining indicated that chondrocyte-derived ECM deposition occurred primarily at the surface of the 2D-ADM scaffolds. In contrast, the 3D-ADM scaffolds exhibited ECM deposition throughout their entire structure (Figure 3C). This observation was further corroborated by Masson’s staining. H&E staining showcased chondrocyte lacunae only at the surface of the 2D-ADM scaffolds, while the 3D-ADM scaffolds demonstrated distributed chondrocyte lacunae (Supplementary Figure S4). Cross-sections subjected to various staining methods reinforced the notion that chondrocyte-derived ECM was predominantly deposited at the edge of the 2D-ADM scaffolds, while the 3D-ADM scaffolds exhibited ECM deposition at both the edge and the center (Supplementary Figure S5).

With a thickness advantage of 1 mm over the 2D-ADM scaffolds, the 3D-ADM scaffolds maintain a cartilage proportion close to 100%, significantly surpassing the 30% observed in the 2D-ADM scaffolds (Figure 3D and G). Moreover, there is a substantial statistical difference in volume exceeding 60 mm³ and wet weight exceeding 80 mg between samples of the 2D- and 3D-ADM scaffolds (Figure 3E and F). Additionally, the content of GAG and type II collagen and their respective ratios to DNA were shown the 3D-ADM scaffold exhibits a DNA content of 178 ng/mg, GAG content of 30 μg/mg and Collagen II content of 14.5 μg/mg, all significantly higher than those of the 2D-ADM scaffolds, with statistical significance observed in all differences (Figure 3H–J and Supplementary Figure S9A and B). These results collectively affirm the superior cartilage regeneration potential of the 3D-ADM scaffolds compared to the 2D-ADM scaffolds. Collectively, these findings highlight the superior full-layer chondrogenic regenerative capacity of 3D-ADM scaffolds compared to the 2D-ADM scaffolds.

### *In vivo* assessment of cartilage regeneration using 2D and 3D-ADM scaffolds

**Figure 4.**
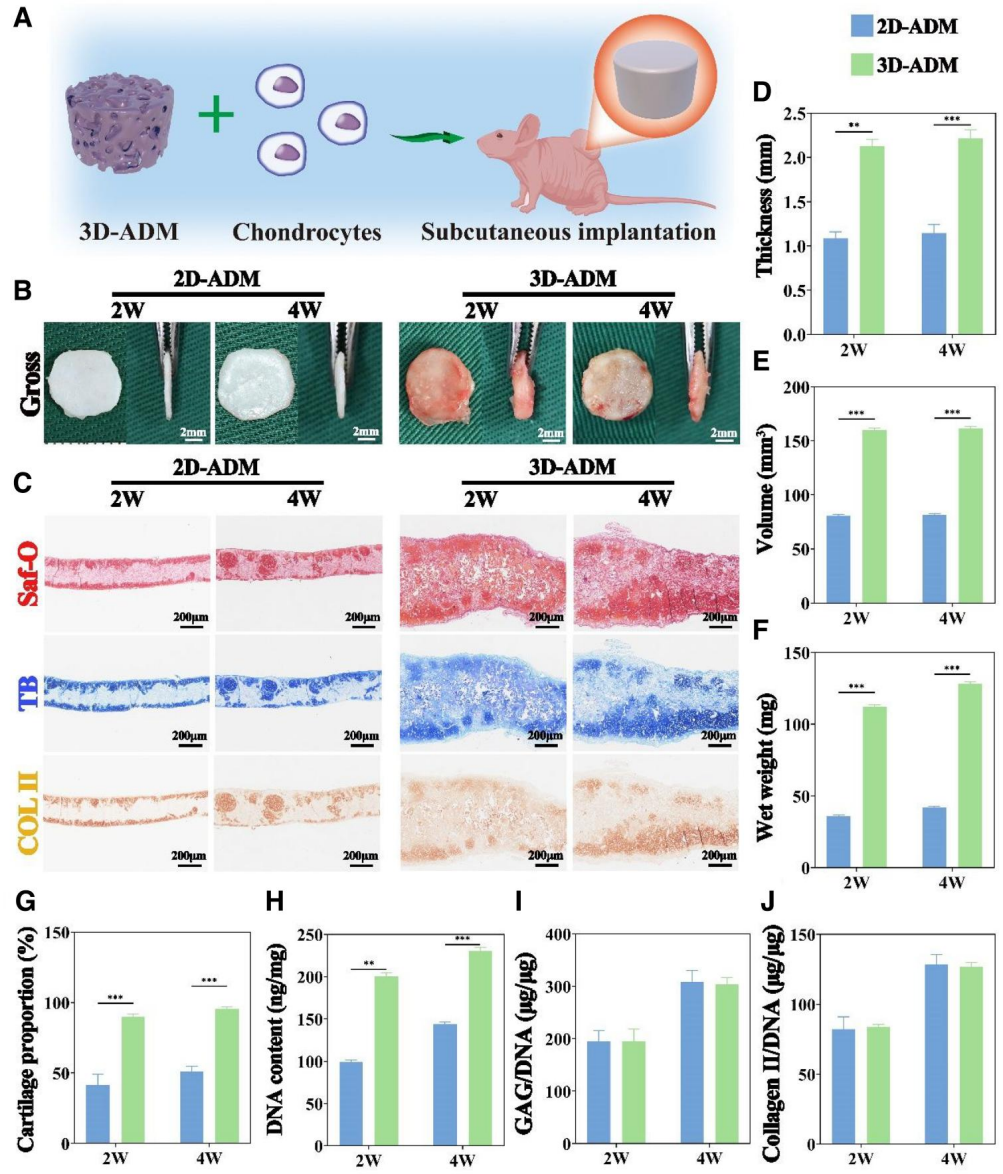
*In vivo* evaluation of cartilage regeneration using 2D- and 3D-ADM scaffolds colonized with chondrocytes after subcutaneous implantation at 2 and 4 weeks. (**A**) Schematic representation of the *in vivo* cartilage regeneration process utilizing 3D-ADM scaffolds colonized with chondrocytes following subcutaneous implantation in nude mice. (**B**) Macroscopic observation of the regenerated cartilage, presented from both top and side perspectives, within 2D- and 3D-ADM scaffolds. (**C**) Staining images showcasing Saf-O, TB and immunohistochemical COL II staining of the regenerated cartilage in 2D- and 3D-ADM scaffolds. Measurement of (**D**) thickness, (**E**) volume, (**F**) wet weight, (**G**) proportion of cartilage, (**H**) DNA content, (**I**) GAG/DNA ratio and (**J**) Collagen II/DNA ratio in the regenerated cartilage note: **P < 0.01 and ***P < 0.00.

To extend the evaluation of chondrogenic regenerative potential, chondrocyte-scaffold constructs were implanted subcutaneously in nude mice and evaluated at 2- and 4-weeks post-implantation (Figure 4A). Macroscopic observation revealed that both scaffold groups retained their initial morphology at both time points. Frontal views indicated that the 2D-ADM scaffolds appeared pale white at 2 weeks and transformed into an ivory white color at 4 weeks, with no evident fiber or vessel adherence on the sample surface. In contrast, the 3D-ADM scaffolds displayed a white-yellow shade at 2 weeks, transitioning into a typical ivory white color at 4 weeks, accompanied by noticeable fiber and small vessel attachments on the sample surface. Lateral views provided further insight, showing that the samples within the 3D-ADM scaffolds were considerably thicker compared to those in the 2D-ADM scaffolds (Figure 4B). Cross-sections of the samples subjected to Saf-O, TB and COL II staining illustrated that chondrocyte-derived ECM deposition occurred primarily at the surface and residual pores remaining after hair follicle removal in the 2D-ADM scaffolds. Conversely, the 3D-ADM scaffolds exhibited widespread deposition of chondrocyte-derived ECM throughout their entire structure (Figure 4C).

Comparative analysis within the same scaffold group revealed that the quantity of chondrocyte-derived ECM deposition at 4 weeks surpassed that at 2 weeks. H&E staining showcased that in both 2D-ADM and 3D-ADM scaffolds, chondrocyte lacunae were primarily concentrated on the surface and residual pores after hair follicle removal at 2 weeks. At 4 weeks, however, the 3D-ADM scaffolds displayed evenly distributed chondrocyte lacunae throughout their entire structure (Supplementary Figure S6). Additional cross-sections stained with H&E reaffirmed that chondrocyte lacunae were localized only at the edge of the 2D-ADM scaffolds, while in the 3D-ADM scaffolds, they were uniformly present both at the edge and center. Saf-O, TB, Masson’s staining and COL II staining further validated the surface and residual pore-centric chondrocyte-derived ECM deposition in the 2D-ADM scaffolds, in contrast to the 3D-ADM scaffolds that exhibited deposition both at the edge and center. Notably, the 3D-ADM scaffolds displayed significant attachment of fibrous layers at the edge, with the thickness of the fibrous caps noticeably increased at 4 weeks (Supplementary Figure S7).

Quantitative analysis of *in vivo* samples reveals that the 3D-ADM scaffold possesses a thickness of 2 mm, a volume of 150 mm³ and wet weights of 110 mg at 2 weeks and 130 mg at 4 weeks, with cartilage deposition approaching nearly 100%. These values are significantly higher than those observed for the 2D-ADM scaffold, with statistically significant results (Figure 4D–G). Additionally, the content of GAG and type II collagen and their respective ratios to DNA were shown at 2 weeks, the DNA content of the 3D-ADM scaffold is 200 ng/mg, GAG content is 40 μg/mg and Collagen II content is 18 μg/mg. At 4 weeks, the DNA content is 230 ng/mg, GAG content is 60 μg/mg and Collagen II content is 26 μg/mg. In comparison to the 2D-ADM scaffold group, these substance levels in the 3D-ADM scaffold are higher at the same cultivation time, with statistically significant differences. Furthermore, as the cultivation time increases, the differences between the two groups become more pronounced (Figure 4H–J and Supplementary Figure S9C and D). These quantitative assessments further demonstrate the superior ability of the 3D-ADM scaffold to promote cartilage growth and proliferation.

### Characterizations and biocompatibility of 3D-ADM+SIS and 3D-ADM+CSH scaffolds

**Figure 5.**
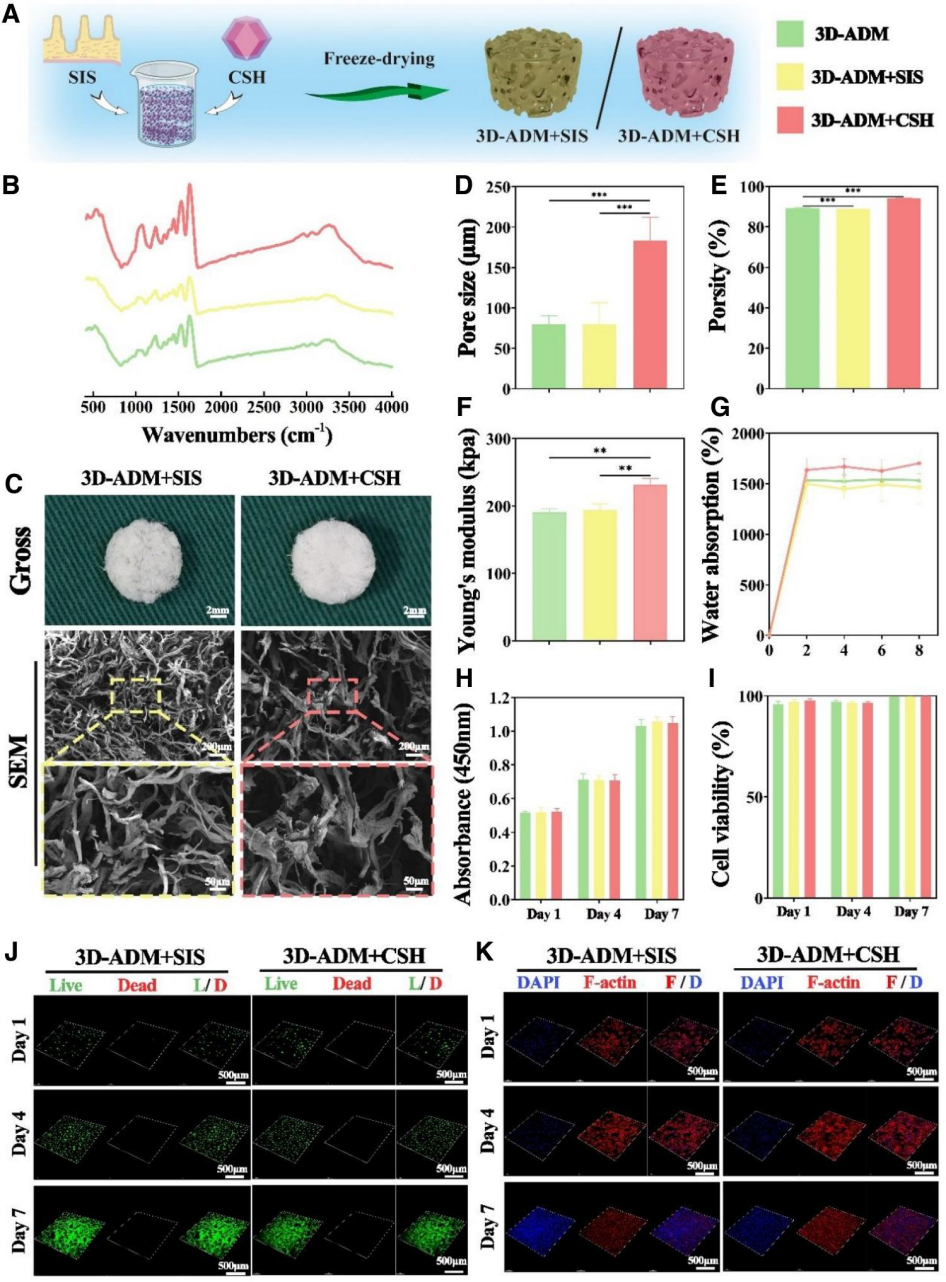
Characterizations and biocompatibility of 3D-ADM+SIS and 3D-ADM+CSH scaffolds. **(A**) Schematic depiction of the process involved in the preparation of bioactive modified 3D-ADM+SIS and biomechanical modified 3D-ADM+CSH scaffolds. (**B**) FTIR spectrum showcasing the comparison between 3D-ADM, 3D-ADM+SIS and 3D-ADM+CSH scaffolds. (**C**) Gross views and SEM images capturing the appearance of 3D-ADM+SIS and 3D-ADM+CSH scaffolds. (**D**) Measurement of pore size in 3D-ADM, 3D-ADM+SIS and 3D-ADM+CSH scaffolds. Assessment of (**E**) porosity, (**F**) Young’s modulus and (**G**) water absorption capability in the scaffolds. (**H**) CCK-8 assay measuring cell proliferation on the scaffolds. (**I**) Evaluation of cell viability in these scaffolds. (**J**) Live & dead cells staining showcasing cell behavior on the scaffolds. (**K**) Visualization of F-actin and DAPI on the scaffolds colonized with chondrocytes after 1–7 days of *in vitro* culture. Note: **P < 0.0 and ***P < 0.001.

Building upon the established chondrogenic regenerative capability of the 3D-ADM scaffolds, further modifications were introduced to enhance scaffold properties like chondrogenic regenerative potential and biomechanical strength. The 3D-ADM+SIS and 3D-ADM+CSH scaffolds were fabricated by incorporating SIS and CSH into the 3D-ADM scaffolds, respectively (Figure 5A). FTIR spectrum analysis depicted absorption peaks at 1100 and 1450 cm^−1^ in the 3D-ADM+CSH scaffold group, indicative of the presence of Ca^2+^ and ${\text{SO}}_{3}^{2-}$, confirming successful loading of CSH. Furthermore, absorption peaks corresponding to -OH (3434 cm^−1^), C-O-C and -NH (1655 and 1560 cm^−1^) and -COO- (1414 cm^−1^) groups were noted in the 3D-ADM+CSH scaffolds, albeit with diminished intensities compared to the 3D-ADM scaffolds. This suggests that the blending of ADM with SIS retained the essential ECM components of ADM (Figure 5B).

Macroscopic examination unveiled uniform and porous cylindrical structures with a porcelain white hue for both the 3D-ADM+SIS scaffolds and the 3D-ADM+CSH scaffolds. SEM images confirmed the presence of abundant pores in both scaffold types (Figure 5C). While the water absorption rates were comparable among 3D-ADM, 3D-ADM+SIS and 3D-ADM+CSH scaffolds, the 3D-ADM+CSH scaffolds displayed the largest pore size, highest porosity and greatest Young’s modulus among the three groups (Figure 5D–G). Live & Dead Cells staining and F-actin/DAPI staining indicated that cells exhibited normal growth, proliferation and spreading on both 3D-ADM+SIS scaffolds and 3D-ADM+CSH scaffolds (Figure 5J and K). Quantitative analysis using CCK-8 and cell viability further supported these observations (Figure 5H and I).

Taken together, these findings confirm the successful combination of SIS and CSH with ADM. Furthermore, they demonstrate that the modified scaffolds do not compromise the satisfactory biocompatibility of the 3D-ADM scaffolds.

### *In vitro* and *in vivo* evaluation of cartilage regeneration using 3D-ADM+SIS and 3D-ADM+CSH scaffolds

**Figure 6.**
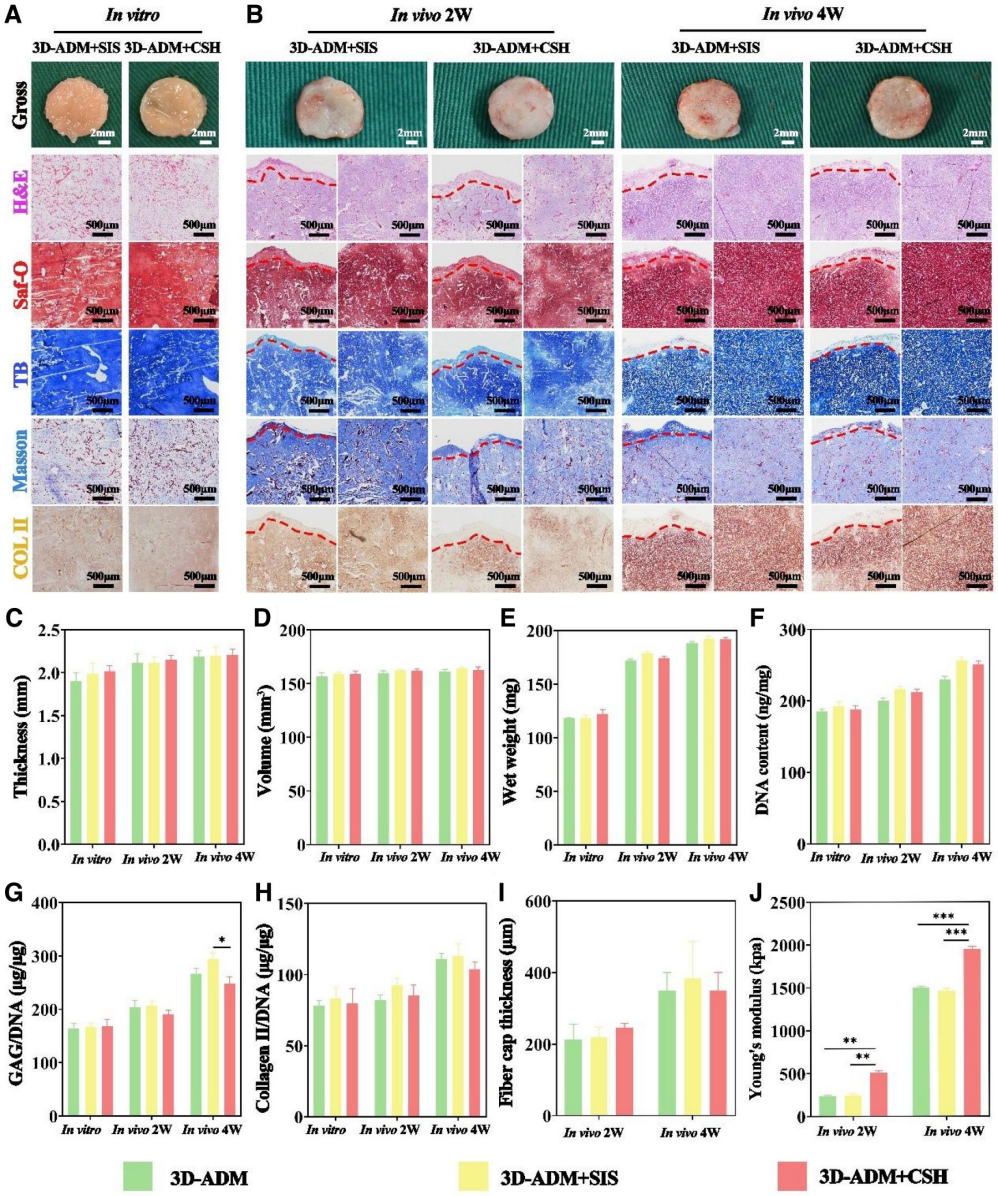
*In vitro* and *in vivo* evaluation of cartilage regeneration using 3D-ADM+SIS and 3D-ADM+CSH scaffolds colonized with chondrocytes. (**A**) Macroscopic views and staining images, including H&E, Saf-O, TB, Masson and immunohistochemical COL II staining, in the 3D-ADM+SIS and 3D-ADM+CSH groups after *in vitro* cultivation for 3 weeks. (**B**) Macroscopic views and staining images, including H&E, Saf-O, TB, Masson and immunohistochemical COL II staining, in the 3D-ADM+SIS and 3D-ADM+CSH groups after subcutaneous implantation in nude mice for 2 and 4 weeks. Measurement of (**C**) thickness, (**D**) wet weight, (**E**) volume, (**F**) DNA content, (**G**) GAG/DNA ratio, (**H**) Collagen II/DNA ratio in the 3D-ADM, 3D-ADM+SIS and 3D-ADM+CSH groups after *in vitro* cultivation for 3 weeks and after subcutaneous implantation in nude mice for 2 and 4 weeks. Measurement of (**I**) fiber cap thickness and (**J**) Young’s modulus in the 3D-ADM, 3D-ADM+SIS and 3D-ADM+CSH scaffolds after subcutaneous implantation in nude mice for 2 and 4 weeks. Note: *P < 0.05, **P < 0.01 and ***P < 0.001.

Following the successful preparation and confirmation of biocompatibility for the 3D-ADM+SIS scaffolds and 3D-ADM+CSH scaffolds, thorough *in vitro* and *in vivo* evaluations of their chondrogenic regenerative potential were conducted. Firstly, after a 3-week *in vitro* culture period, macroscopic observations indicated a bright yellow-white color for both scaffold types. The 3D-ADM+SIS scaffolds retained their morphology well, while the 3D-ADM+CSH scaffolds maintained their cylindrical shape intact. H&E staining unveiled rich and uniform cartilage-specific lacunae in both scaffold groups. Additional staining methods, such as Saf-O, TB, Masson’s trichrome and COL II immunohistochemical staining, confirmed the deposition of cartilage-specific ECM in both types of scaffolds (Figure 6A).

Secondly, after culturing in nude mice for 2 and 4 weeks, macroscopic assessments demonstrated that at 2 weeks, both scaffold groups appeared porcelain white with slight fiber and vascular attachment on the surface. The cylindrical morphology of the 3D-ADM+CSH scaffolds was notably well-maintained. At 4 weeks, both groups transitioned to the typical ivory white color with increased fiber and vascular attachment on the surface, while retaining their original morphology (Figure 6B).

H&E staining results showed abundant and uniform cartilage-specific lacunae as well as some fiber tissue attachment in both scaffold groups at both time points. Staining with Saf-O, TB, Masson’s trichrome and immunohistochemical COL II staining confirmed abundant deposition of cartilage-specific ECM in both scaffold groups. A comparison within the same group between different time points highlighted increased deposition of cartilage-specific ECM and thicker fiber caps at 4 weeks. In comparison between the two scaffold types at the same time point, there was no substantial variation in cartilage-specific ECM. Furthermore, at 2 weeks, the fibrous cap thickness in both groups of scaffolds was similar. At 4 weeks, the fibrous cap of the 3D-ADM+SIS scaffolds was slightly thicker than that of the 3D-ADM+CSH scaffolds, but the difference was not significant (Figure 6B). Further magnified observation of the edges and centers of stained images from the repair site provided additional confirmation (Supplementary Figure S8).

Quantitative analysis reveals similar data for 3D-ADM, 3D-ADM+SIS and 3D-ADM+CSH scaffolds during *in vitro* cultivation, with no statistically significant differences among groups. Specimens in each group exhibit ∼2 mm thickness, a volume of around 150 mm³ and a wet weight of 120 mg. The content of DNA, GAG and Collagen II and their respective ratios to DNA were shown with DNA, GAG and Collagen II content at 190 ng/mg, 30 and 15 μg/mg, respectively. After 2 weeks of *in vivo* cultivation, specimens in each group show thickness and volume similar to those *in vitro*, with a wet weight of around 170 mg, a fibrous cap thickness of 200 μm and comparable DNA and Collagen II content at 200 ng/mg and 40 μg/mg. Notably, the Collagen II content in the 3D-ADM+SIS scaffold is significantly higher, ∼20 μg/mg, compared to the 3D-ADM scaffold (*P* < 0.05), and the Young’s modulus of the 3D-ADM+CSH scaffolds is the highest at 500 kPa, significantly different from the other two groups (*P* < 0.01). After 4 weeks of *in vivo* cultivation, changes in thickness and volume are not substantial, with a slight increase in wet weight, but a significant increase in DNA, GAG and Collagen II content. It is worth noting that the GAG content in the 3D-ADM+SIS scaffolds is the highest, ∼70 μg/mg, significantly different from the 3D-ADM+CSH scaffolds (*P* < 0.05). The Young’s modulus of the 3D-ADM+CSH scaffold remains the highest, approaching 2000 kPa, with a further significant difference from the other two groups (*P* < 0.001) (Figure 6C–J and Supplementary Figure S9E and F). Although not significant, but in each group can achieve good cartilage regeneration premise, the 3D-ADM+SIS scaffolds exhibited higher GAG and Collagen II content and greater fibrous cap thickness compared to the other two groups, it was revealed that the bioactive modified 3D-ADM+SIS scaffolds exhibited enhanced chondrogenic regenerative effects and improved biointegration ability to a certain extent. In contrast, the biomechanically modified 3D-ADM+CSH scaffolds notably enhanced the mechanical properties of the regenerated cartilage.

### *In situ* evaluation of articular cartilage repair in a rabbit model

**Figure 7.**
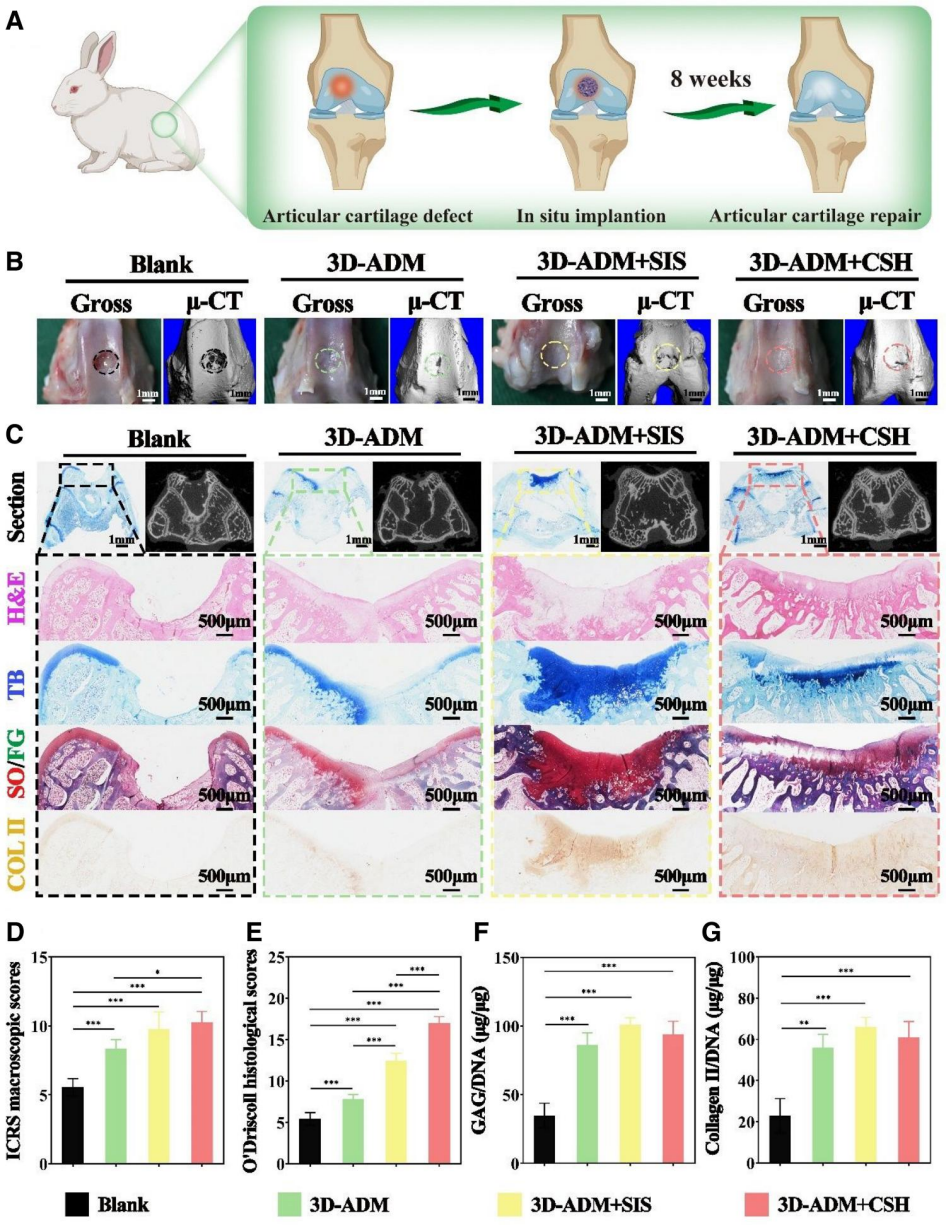
*In situ* evaluation of cartilage repair using 3D-ADM, 3D-ADM+SIS and 3D-ADM+CSH scaffolds in a rabbit articular cartilage defect model for 8 weeks. (**A**) Schematic representation illustrating the process of *in situ* cartilage repair using a rabbit articular cartilage defect model. (**B**) Macroscopic and micro-CT observation images depicting the outcomes in the blank, 3D-ADM, 3D-ADM+SIS and 3D-ADM+CSH groups. (**C**) Staining images including H&E, TB, so/FG and immunohistochemical COL II staining, showcasing the results in the same groups. (**D**) Evaluation of ICRS macroscopic scores. (**E**) Assessment of O’Driscoll histological scores. Determination of (**F**) GAG/DNA and (**G**) Collagen II/DNA ratio in the same groups. Note: *P < 0.05, **P < 0.01 and ***P < 0.001.

After confirming the physical characteristics, biocompatibility and chondrogenic regenerative performance of the various scaffold types, the next crucial step involves *in situ* implantation to facilitate cartilage regeneration (Figure 7A). The blank, 3D-ADM, 3D-ADM+SIS and 3D-ADM+CSH groups were implanted into rabbit articular cartilage defect models for *in situ* repair, and samples were collected after 8 weeks for evaluation. Macroscopic observation and micro-CT 3D reconstructions revealed significant defects in the blank group. In contrast, the articular cartilage defects in the 3D-ADM, 3D-ADM+SIS and 3D-ADM+CSH groups had undergone partial repair, with the 3D-ADM+SIS and 3D-ADM+CSH groups exhibiting superior outcomes (Figure 7B). ICRS macroscopic scores criteria revealed scores of 10 for both the 3D-ADM+SIS and 3D-ADM+CSH groups, significantly higher than the score of eight for the 3D-ADM group (*P* < 0.01) and the score of five for the blank group (*P* < 0.001). The difference in scores between the 3D-ADM group and the blank group was also highly significant (*P* < 0.001). This indicates that the 3D-ADM scaffold possesses a certain degree of joint defect repair capability, with the cartilage repair effects of the 3D-ADM+SIS and 3D-ADM+CSH scaffolds proving more effective (Figure 7D).

H&E staining depicted minimal cartilage regeneration in the blank group, predominantly fiber tissue filling in the joint defects of the 3D-ADM group, the most extensive layer of regenerated cartilage in the 3D-ADM+SIS group and joint repair with the 3D-ADM+CSH group closely resembling normal morphology. Staining with SO/FG, TB and immunohistochemical COL II staining revealed minimal deposition of cartilage-specific ECM in the blank group, limited deposition in the joint defects of the 3D-ADM group, the highest level of cartilage-specific ECM deposition in the 3D-ADM+SIS group and joint repair with the 3D-ADM+CSH group approaching the deposition state of normal joints. Additionally, lateral views of the defect area from micro-CT indicated minimal regeneration of subchondral bone tissue in the blank group, moderate regeneration in the joints repaired with the 3D-ADM scaffold and 3D-ADM+SIS scaffolds and the most effective subchondral bone regeneration in the 3D-ADM+CSH scaffolds (Figure 7C).

O'Driscoll histological scoring demonstrated the highest score of 17 for the 3D-ADM+CSH group, followed by a slightly lower score of 13 for the 3D-ADM+SIS group, where the majority of the filling tissue was cartilage. The 3D-ADM group, filled predominantly with fibrous tissue, scored eight, while the blank group scored five. The differences among the groups were statistically significant (*P* < 0.001) (Figure 7E). Finally, the content of GAG and Collagen II and their respective ratios to DNA were shown that the 3D-ADM+SIS group had the highest levels, at 0.9 and 1.2 μg/mg, slightly surpassing the 0.75 and 1.1 μg/mg in the 3D-ADM+CSH group, with no statistically significant difference. However, the content in both the 3D-ADM+SIS and 3D-ADM+CSH groups was significantly higher than that in the 3D-ADM and blank groups, with statistically significant differences in the results (Figure 7F and G and Supplementary Figure S9G and H). These results indicate better cartilage regeneration effects for the modified 3D-ADM+SIS and 3D-ADM+CSH scaffolds, confirming the success of our modification strategy.

## Discussion

Cartilage tissue engineering commonly relies on cartilage as the primary source. For instance, Gong *et al.* [34] achieved successful rabbit knee cartilage defect repair by using a 3D-ACM scaffold modified with graphene oxide. Jia *et al.* [35] improved the repair of articular cartilage defects by combining ACM with monocytes. Despite its widespread use, the dense structure of cartilage makes complete cell removal challenging, and the processing steps are complex. Additionally, the delicate ultrastructure and nutritional factors can be easily damaged, and cartilage availability is limited [36]. As a result, researchers are exploring alternative DEM for cartilage regeneration. For example, Gao *et al.* [10] achieved tracheal cartilage regeneration using a loosely structured umbilical cord decellularized matrix hydrogel.

Among various decellularized materials, ADM stands out due to its wide availability, affordability, loose structure and strong biological activity [37]. Importantly, studies have shown that ADM outperforms ACM in terms of cartilage regeneration [16]. Consequently, ADM has gained substantial attention in the cartilage tissue engineering field. For instance, Lei and Huang [38] successfully employed ADM for the restoration of nasal cartilage post-tumor excision. Meanwhile, Ye *et al.* [39] achieved a significant reparative outcome in mending articular cartilage defects in rabbit knee joints by administering orally administered naringin in conjunction with ADM. However, creating a 3D tissue engineering scaffold from 2D-ADM with suitable pore size, uniform porosity, high mechanical strength and controllable shape remains a hurdle.

This study introduces an innovative decellularization method to prepare 2D-ADM scaffolds, which are then transformed into 3D-ADM scaffolds through a novel series of steps. Initially, a combination of chemical and enzymatic decellularization methods was found to efficiently and completely remove cells, with DNA content (20 ng/mg) significantly below the 50 ng/mg immunoreaction threshold. The quantitative detection of total collagen content and qualitative analysis of elastic fibers indicated that the ECM components of the dermis remained largely intact following decellularization. This success underscores the effectiveness of the employed decellularization method.

Subsequent to the successful transformation of 2D-ADM scaffolds into 3D configurations, favorable attributes, such as appropriate pore size, porosity, water absorption and mechanical strength were achieved. These features are conducive to cellular adhesion, growth and proliferation. Biocompatibility assessments revealed that the preparation of ADM and its 3D transformation effectively preserve the bioactive factors inherent in the dermis. The porous 3D architecture of 3D-ADM scaffolds facilitates chondrocyte adhesion, diffusion and proliferation throughout the scaffold, in contrast to the limited surface cell growth observed with 2D-ADM scaffolds. This underscores the imperative shift from 2D to 3D structures for efficient cartilage regeneration and emphasizes the outstanding biocompatibility of ADM.

Both 2D-ADM scaffolds and 3D-ADM scaffolds demonstrated cartilage adhesion on their surfaces *in vitro* for 3 weeks. Subsequent assessments unveiled distinct characteristics between the two, with experimental outcomes consistently indicating superior cartilage regeneration efficacy for the 3D-ADM scaffold compared to its 2D counterpart. The regenerative potential of both scaffolds can be attributed to ADM’s nutritional constituents, bioactivity and ultrastructure, which can partly emulate natural cartilage. Nonetheless, the limitations of 2D-ADM scaffolds, including small pore size, low porosity and inadequate water absorption, restrict regenerated cartilage to the surface and impede inward growth. On the contrary, the transition from 2D-ADM to 3D-ADM scaffolds introduces ample internal pores, an appropriate pore size and suitable mechanical strength, facilitating comprehensive-layer cartilage regeneration. When applied to *in situ* joint injury repair, 3D-ADM scaffolds demonstrated some reparative effects. However, we observed disparities in both the biological activity and mechanical strength between cartilage regenerated *in vitro* and *in vivo* using the 3D-ADM scaffolds when compared to natural cartilage. Furthermore, the articular *in situ* repair site of the 3D-ADM scaffolds is predominantly composed of fibrous tissue, and the outcomes of regenerated cartilage are not entirely satisfactory. To overcome this limitation, several approaches can be adopted, including (i) cell therapy: e.g. Jiang *et al.* [40] demonstrated a method of injecting BMSCs into decellularized umbilical cord matrix scaffolds, which promoted the regeneration of bone cartilage; (ii) increasing the concentration of growth factors: for instance, Huang *et al.* [41] showed that the addition of concentrated growth factors could enhance the effectiveness of bone cartilage regeneration; (iii) modifying scaffold fabrication methods or processes, such as 3D printing; and (iv) direct modification of ADM scaffolds to improve their efficacy in cartilage regeneration. Among various approaches, direct modification of scaffolds offers greater customization and controllability, leading to significantly improved bioactivity and biomechanics of the scaffold, thereby enhancing the effectiveness of cartilage regeneration. Therefore, we aim to enhance the bioactivity and biomechanics of 3D-ADM scaffolds by incorporating different modifying agents.

In prior investigations, we have ascertained that decellularized SIS serves as an exceptional matrix material following decellularization [42]. SIS demonstrates remarkable biocompatibility and low immunogenicity. It facilitates tissue repair devoid of scarring or calcification, and is rich in growth factors including TGF-β, b-FGF and VEGF [43]. These attributes have rendered SIS widely applicable across diverse domains, such as blood vessels, trachea, skin and tendons [44–46]. However, the absence of vital ECM constituents, coupled with intrinsic microstructural heterogeneity and limited porosity, constrains its utility within the cartilage tissue engineering domain [47]. Notably, the growth factors like TGF-β, b-FGF and VEGF inherent to SIS play a pivotal role in cartilage regeneration. Encouragingly, numerous studies have substantiated the presence of abundant collagen, elastic fibers, proteoglycans, growth factors and ECM within ADM [16, 48]. The presence of the aforementioned substances establishes a robust foundation for the modification of 3D-ADM scaffolds with SIS.

Given the potential synergies between SIS and ADM, our study integrated SIS into ADM, successfully fabricating the 3D-ADM+SIS scaffold. This scaffold possesses appropriate physicochemical properties, excellent biocompatibility and demonstrates superior *in vitro* and *in vivo* cartilage regeneration compared to the 3D-ADM scaffolds. Most notably, the tissues post *in situ* repair of the knee joint are primarily composed of cartilage, providing compelling evidence for the robust *in situ* cartilage regeneration capability of the 3D-ADM+SIS scaffolds. We posit that the enhanced outcomes observed in cartilage regeneration employing 3D-ADM+SIS scaffolds stem primarily from the synergistic interplay of ECM components and growth factors found in both ADM and SIS. This synergy fosters chondrocyte growth and proliferation, facilitating the deposition of cartilage lacunae and cartilage-specific ECM. Additionally, capitalizing on ADM’s efficacy as a medium and the innovative scaffold preparation technique, the incorporation of SIS addresses the challenges related to SIS’s inherent microstructural heterogeneity and limited porosity. Thus, our investigation confirms that the strategy of fabricating 3D-ADM+SIS scaffolds by integrating SIS into ADM stands as a more advantageous approach for cartilage regeneration. This approach not only upholds the exceptional attributes of 3D-ADM scaffolds but also engenders a synergistic effect that surpasses the cumulative impact of its individual components.

Having achieved biologically active modification of the 3D-ADM scaffolds through the introduction of SIS, further enhancements can be achieved by performing biomechanical modifications on these scaffolds. CSH stands out as a highly osteoconductive bone substitute extensively employed in clinical settings. Renowned for its robust mechanical strength and biocompatibility, CSH is a common choice as a scaffold material for bone tissue regeneration in tissue engineering [49]. For instance, Cao *et al.* [28] developed an injectable hydrogel for accelerated regeneration of cartilage and subchondral bone by grafting carbonyl hydrazide-modified collagen with CSH. Chang *et al.* [50] developed a novel composite material by incorporating strontium-doped CSH and hydroxyapatite, facilitating bone formation through recruitment and stimulation of osteogenic differentiation of bone marrow mesenchymal stem cells. Importantly, they noted the presence of chondrocytes within the composite material’s pores. In alignment with these findings, our study encompassed the integration of CSH into ADM to fabricate the 3D-ADM+CSH scaffolds.

Considering the robust biomechanical effects introduced by CSH, our study integrated CSH into ADM, successfully fabricating the 3D-ADM+CSH scaffold. In comparison to both 3D-ADM and 3D-ADM+SIS scaffolds, this scaffold exhibited superior mechanical properties while maintaining excellent biocompatibility. Furthermore, the biomechanical status of cartilage regenerated *in vitro* and *in vivo* using this scaffold closely resembled that of natural cartilage. Notably, in *in vivo* experiments for rabbit knee joint defect repair, the 3D-ADM+CSH scaffold achieved a repair outcome that closely approximated the morphological characteristics of natural joint cartilage. These positive outcomes can be primarily attributed to the substantial reinforcement in scaffold strength resulting from CSH incorporation. Furthermore, the exceptional biocompatibility and bioinductivity of CSH facilitated the retention of ADM’s ECM components and bioactive factors within the 3D-ADM+CSH scaffolds. This synergy, coupled with improved scaffold mechanical attributes, further fostered the development of cartilage lacunae and the deposition of cartilage-specific ECM. Lastly, as CSH aids in the induction and differentiation of bone marrow mesenchymal stem cells into chondrocytes and osteoblasts, the *in situ* repair outcomes using the 3D-ADM+CSH scaffolds closely mirrored the morphological features of a healthy joint. Thus, our investigation affirms that the strategy of integrating CSH into ADM for the preparation of 3D-ADM+CSH scaffolds not only preserves the exceptional attributes of 3D-ADM scaffolds but also augments the biomechanical performance of regenerated cartilage and the effectiveness of articular cartilage regeneration.

## Conclusion

In this study, we have introduced an innovative decellularization method to prepare ADM and subsequently transform 2D-ADM into 3D scaffolds. These scaffolds showcase favorable physicochemical attributes, exceptional biocompatibility and substantial potential for driving cartilage regeneration both *in vitro* and *in vivo*. To amplify the cartilage regeneration and repair potential of 3D-ADM scaffolds, we undertook further enhancements by integrating porcine-derived SIS for bioactivity and CSH for biomechanical fortification. The 3D-ADM+SIS scaffolds exhibited heightened biological activity, while the 3D-ADM+CSH scaffolds significantly bolstered biomechanical strength, for cartilage regeneration and repair in both laboratory and animal settings. Notably, these modifications exhibited considerable improvements in repairing cartilage defects within a rabbit articular cartilage model. In conclusion, this research has introduced a versatile 3D-ADM scaffold with customizable bioactive and biomechanical attributes. Promisingly, this holds the potential to offer novel insights into the field of cartilage regeneration.

## Supplementary Material

rbae010_Supplementary_Data
