# Supplementary material for: Enhancing cartilage regeneration and repair through bioactive and biomechanical modification of 3D acellular dermal matrix
Source: Regen Biomater. 2024 Feb 5;11:rbae010. doi: 10.1093/rb/rbae010 (PMC10898337; doi:10.1093/rb/rbae010)
Supplement: rbae010_Supplementary_Data [file rbae010_supplementary_data.docx]

**Supporting Information**

**Enhancing Cartilage Regeneration and Repair through Bioactive and Biomechanical Modification of 3D Acellular Dermal Matrix**

Wei Gao^1a^, Tan Cheng^9a^, Zhengya Tang^8a^, Wenqiang Zhang^7^, Yong Xu^10^, Min Han^4^, Guangdong Zhou^8^, Chunsheng Tao^6*^, Ning Xu^3,4*^, Huitang Xia^5*^, Weijie Sun^2*^

^1^ Medical School of Qingdao University, Qingdao, China

^2^ Department of Infectious Diseases, The First Affiliated Hospital of Anhui Medical University, Shushan, Hefei, China

^3^ Department of Orthopaedic Surgery, Shanghai Sixth People's Hospital Affiliated to Shanghai Jiao Tong University School of Medicine, Shanghai, China

^4^ Department of Orthopedic Surgery, Shanghai Eighth People's Hospital, Shanghai, China

^5^ Department of Plastic Surgery & Jinan Clinical Research Center for Tissue Engineering Skin Regeneration and Wound Repair, The First Affiliated Hospital of Shandong First Medical University, Jinan, China

^6^ Department of Orthopaedics, Ninety-seventh Hospital of the Chinese People's Liberation Army Navy, Qingdao, China

^7^ Department of Orthopaedics, The First Affiliated Hospital of Shandong First Medical University, Jinan, China

^8^ Shanghai Key Laboratory of Tissue Engineering, Shanghai Ninth People’s Hospital, Shanghai Jiao Tong University School of Medicine, Shanghai, China

^9^ Department of Cardiothoracic Surgery, Shanghai Children’s Hospital, Shanghai Jiao Tong University School of Medicine, Shanghai, China

^10^ Department of Thoracic Surgery, Shanghai Pulmonary Hospital, School of Medicine, Tongji University, Shanghai, China

^a^ These authors contributed equal to this work.

* Corresponding authors: Dr. Chunsheng Tao, Email: doctortao2008@163.com; Dr. Ning Xu, Email: ixuning@163.com; Prof. Huitang Xia, Email: [xiahuitang@163.com](mailto:xiahuitang@163.com); Dr. Weijie Sun, Email: swj950128@163.com.


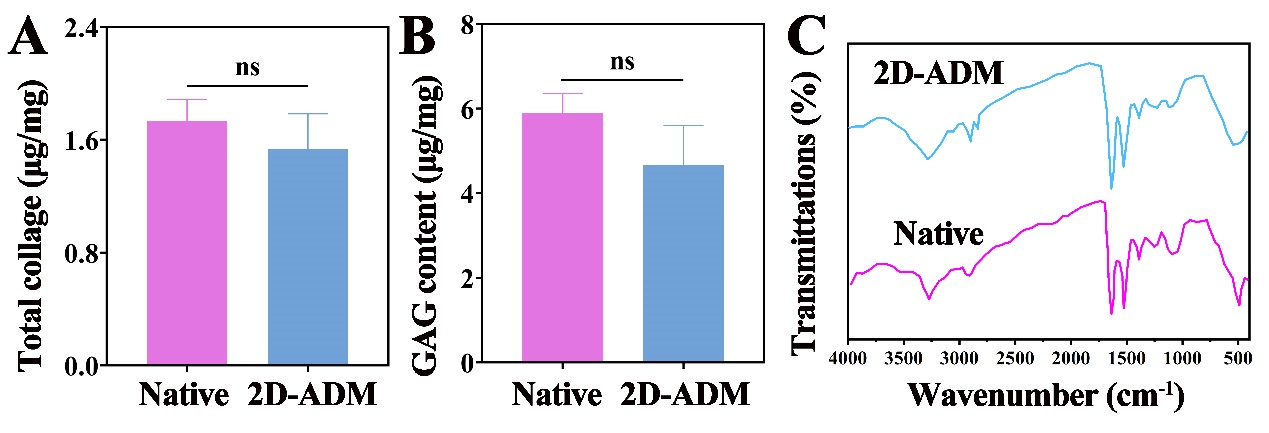


**Figure S1:** **Qualitative determination of total collagen content, GAG content and FTIR of elastin in natural dermis and 2D-ADM.**


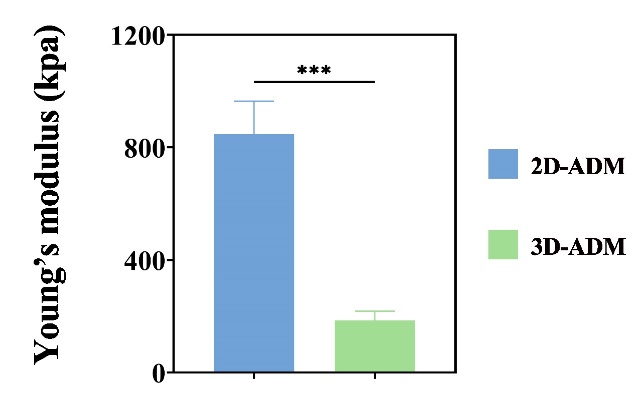


**Figure S2:** **Young's modulus of the 2D- and 3D-ADM scaffolds.**


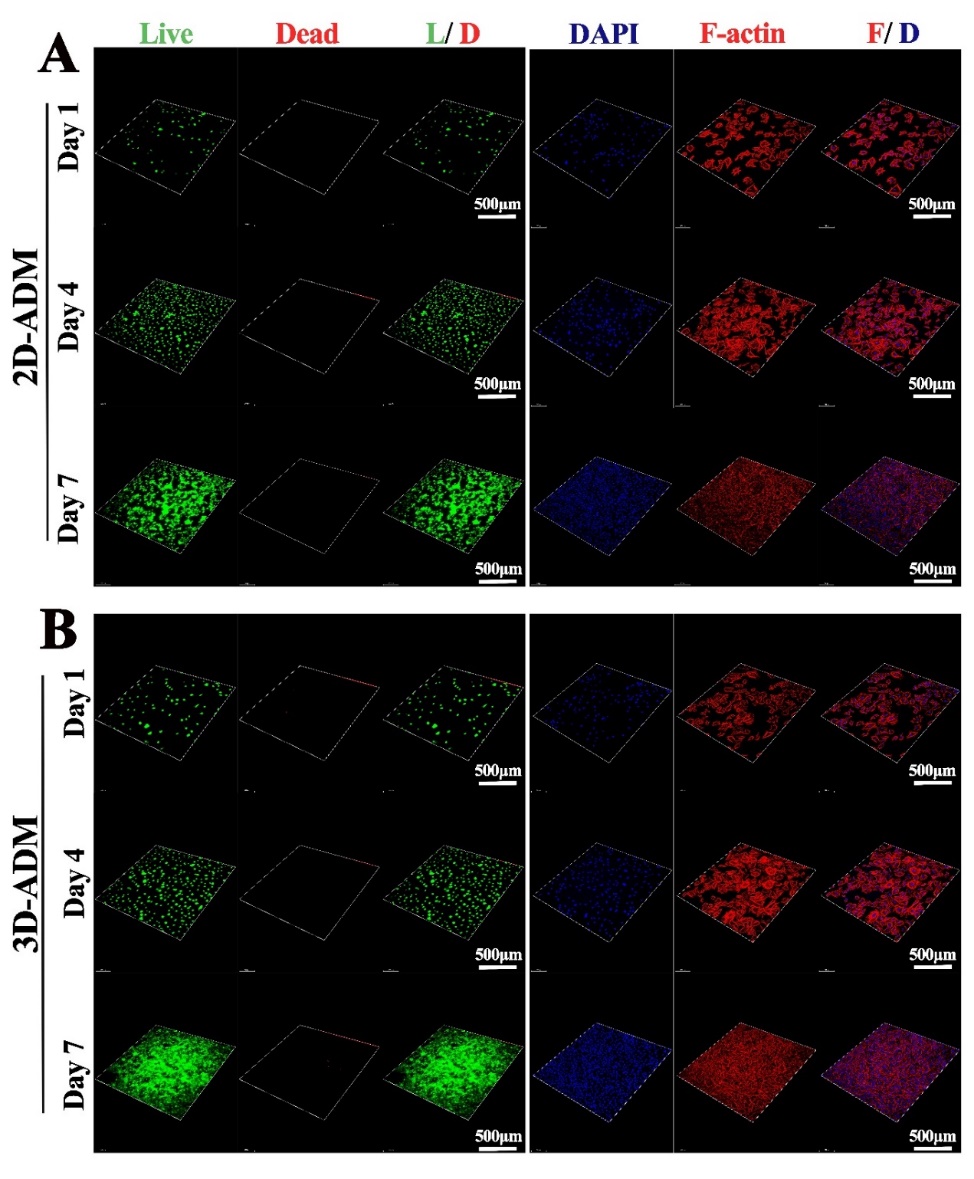


**Figure S3: Live & Dead Cells staining and F-actin/DAPI staining of the 2D- and 3D-ADM scaffolds after *in vitro* cocu1tured with chondrocytes for 1-7 days.**

**
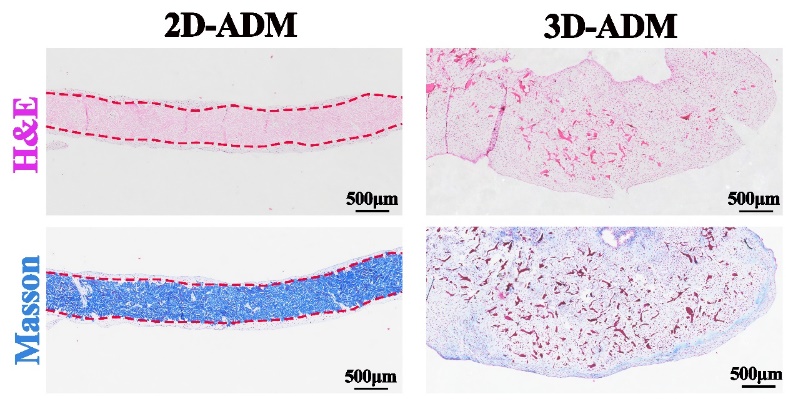
**

**Figure S4: H&E and Masson Staining of *in vitro* regenerated cartilage using the 2D- and 3D-ADM scaffolds colonized with chondrocytes for 3 weeks on side view.**


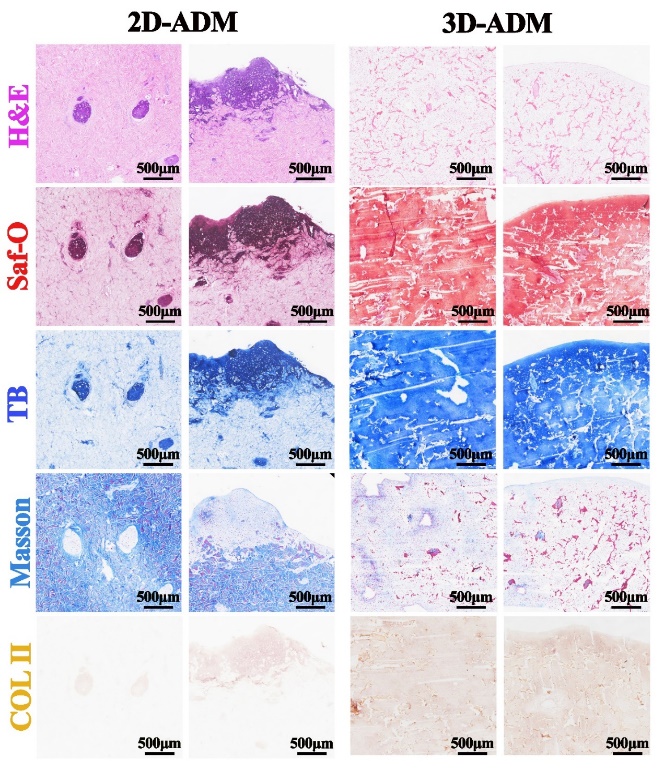


**Figure S5: H&E, Saf-O, TB, Masson, and immunohistochemical COL II Staining of *in vitro* regenerated cartilage using the 2D- and 3D-ADM scaffolds colonized with chondrocytes for 3 weeks on top view.**

**
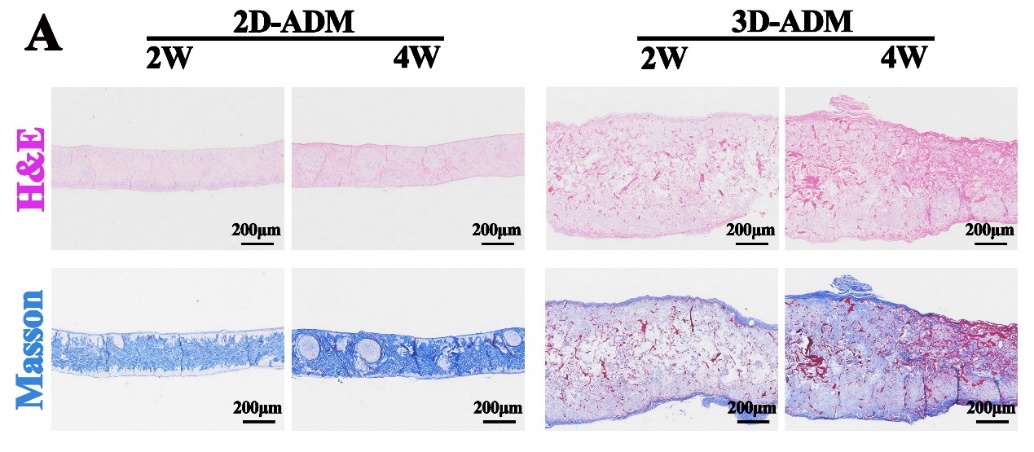
**

**Figure S6: H&E and Masson Staining of regenerated cartilage using 2D- and 3D-ADM scaffolds colonized with chondrocytes after subcutaneous implantation at 2 and 4 weeks on side view.**

**
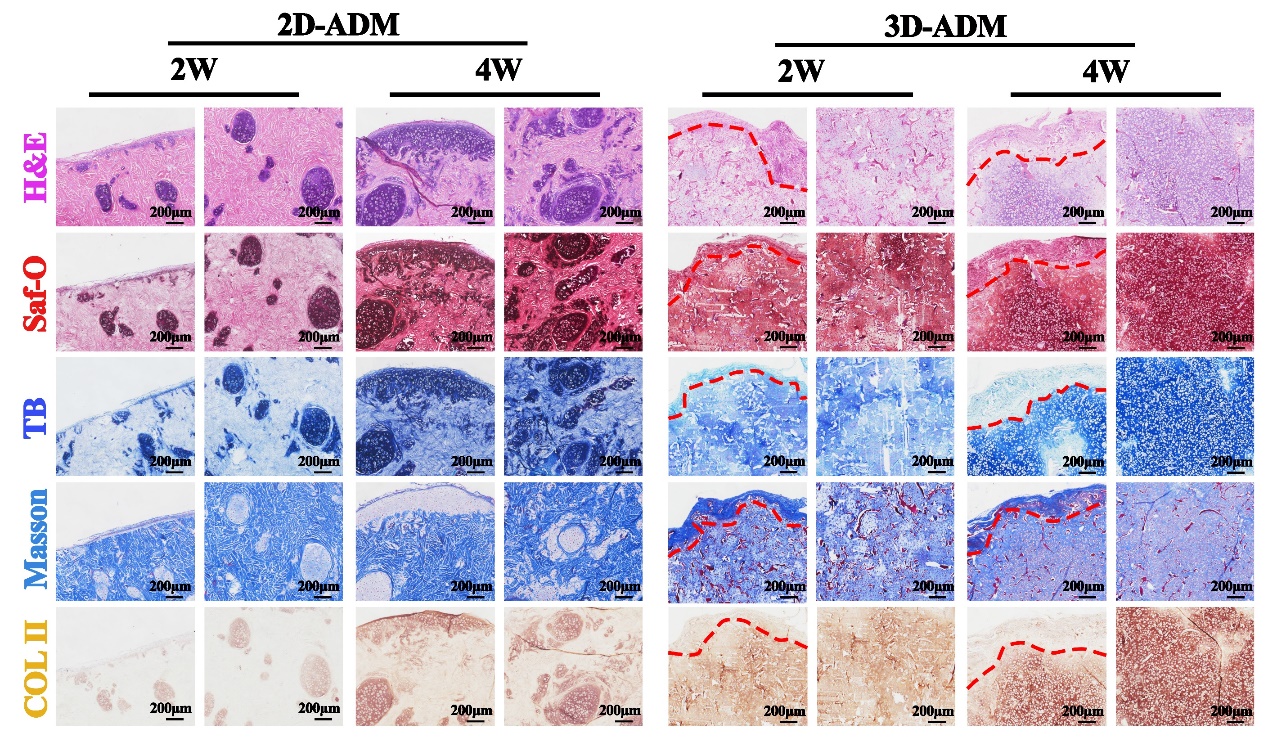
**

**Figure S7: H&E, Saf-O, TB, Masson and immunohistochemical COL II Staining of regenerated cartilage using 2D- and 3D-ADM scaffolds colonized with chondrocytes after subcutaneous implantation at 2 and 4 weeks on top view.**

**
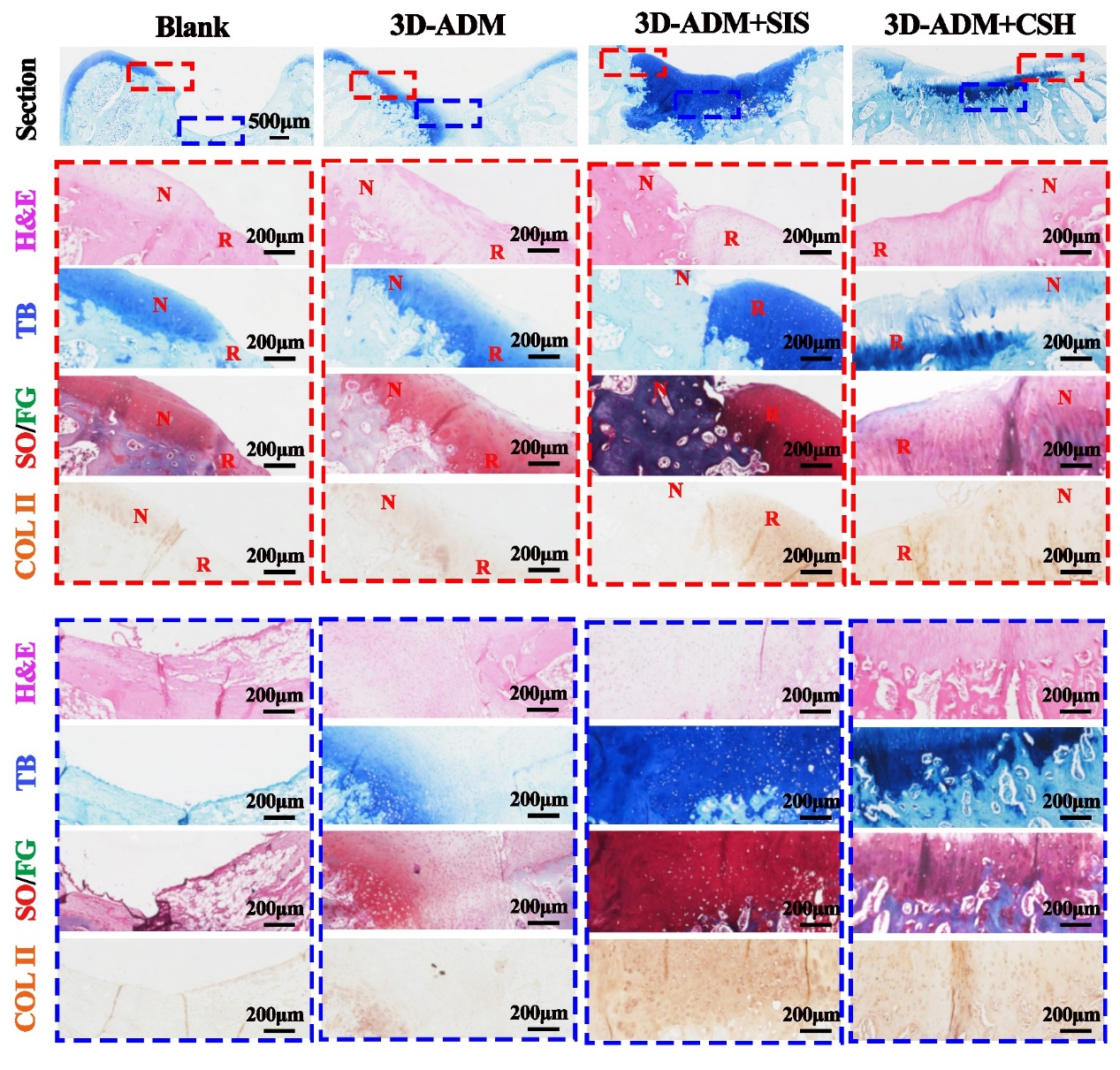
**

**Figure S8: Enlarged View of H&E, TB, SO/FG and immunohistochemical COL II Staining of the repaired articular cartilage center and edge in Blank, 3D-ADM, 3D-ADM+SIS and 3D-ADM+CSH groups in rabbit articular cartilage defect models for 8 weeks.**

**
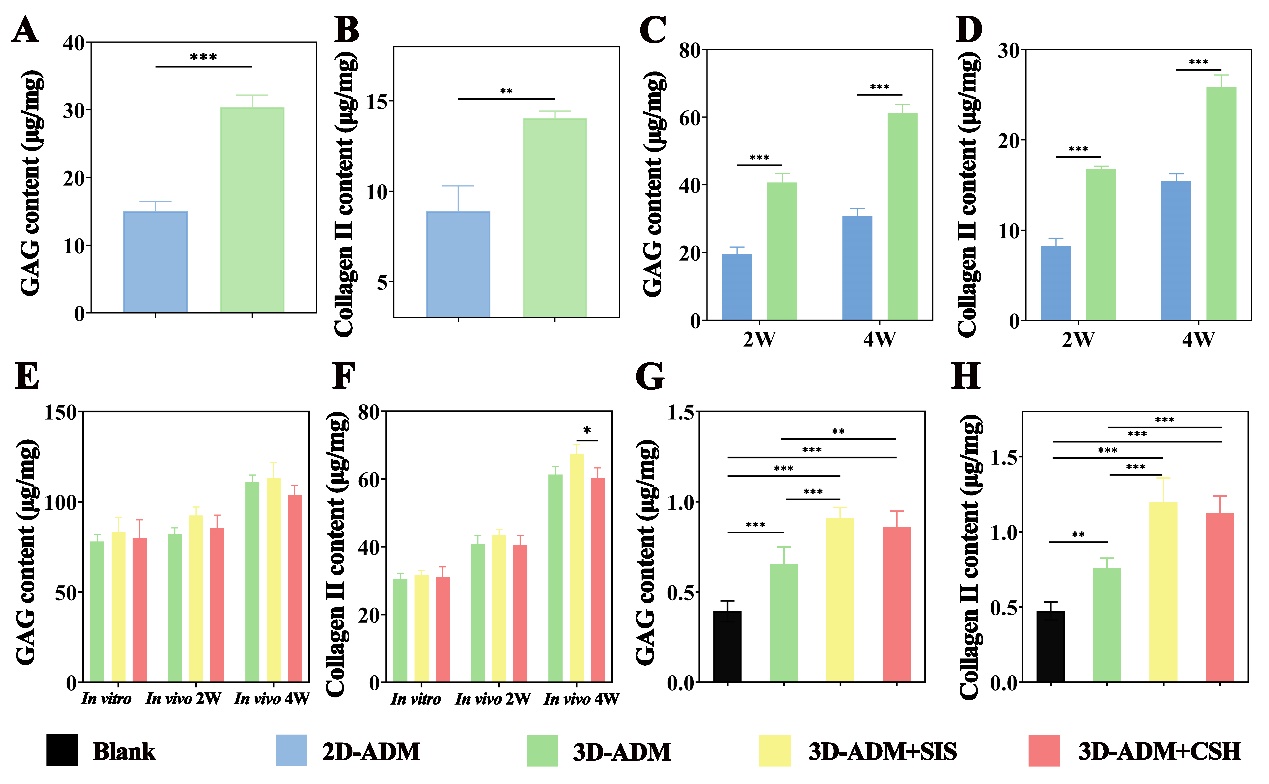
**

**Figure S9:** **In vitro, in vivo, and in situ GAG and Collagen II contents were evaluated for 3D-ADM, 3D-ADM+SIS, and 3D-ADM+CSH scaffolds.**

**Supplementary Table 1. The ICRS macroscopic scoring system**

| **Feature** | **Score** |
| --- | --- |
| Coverage |  |
| >75% fill | 4 |
| 50-75% fill | 3 |
| 25-50% fill | 2 |
| <25% fill | 1 |
| No fill | 0 |
| Neocartilage color |  |
| Normal | 4 |
| 25% yellow/brown | 3 |
| 50% yellow/brown | 2 |
| 75% yellow/brown | 1 |
| 100% yellow/brown | 0 |
| Defect margins |  |
| Invisible | 4 |
| 25% circumference visible | 3 |
| 50% circumference visible | 2 |
| 75% circumference visible | 1 |
| 100% circumference visible | 0 |
| Overall repair assessment |  |
| Grade I: normal | 12 |
| Grade II: nearly normal | 11-8 |
| Grade III: abnormal | 7-4 |
| Grade IV: severely abnormal | 3-1 |

This table was adopted from Ref. (Histological assessment of cartilage repair: a report by the Histology Endpoint Committee of the International Cartilage Repair Society (ICRS))

**Supplementary Table 2. O'Driscoll histological scoring system**

| **Feature** | **Score** |
| --- | --- |
| (a) Nature of the predominant tissue |  |
| 1 Cellular morphology | 4 |
| Hyaline articular cartilage | 2 |
| Incompletely differentiated mesenchyme | 0 |
| 2 Safranin-O staining of the matrix |  |
| Normal or nearly normal | 3 |
| Moderate | 2 |
| Slight | 1 |
| None | 0 |
| (b) Structural characteristics | 3 |
| 3 Surface regularity |  |
| Smooth and intact | 3 |
| Superficial horizontal lamination | 2 |
| Fissures - 25 to 100 per cent of the thickness | 1 |
| Severe disruption. including fibrillation | 0 |
| 4 Structural integrity |  |
| Normal | 2 |
| Slight disruption. including cysts | 1 |
| Severe disintegration | 0 |
| 5 Thickness |  |
| 100 per cent of normal adjacent cartilage | 2 |
| 50-100 per cent of normal cartilage | 1 |
| 0-50 per cent of normal cartilage | 0 |
| 6 Bonding to the adjacent cartilage |  |
| Bonded at both ends of graft | 2 |
| Bonded at one end, or partially at both ends | 1 |
| Not bonded | 0 |
| (c) Freedom from cellular changes of degeneration |  |
| 7 Hypocellularity |  |
| Normal cellularity | 3 |
| Slight hypocellularity | 2 |
| Moderate hypocellularity | 1 |
| Severe hypocellularity | 0 |
| 8 Chondrocyte clustering |  |
| No clusters | 2 |
| <25 per cent of the cells | 1 |
| 25-100 per cent of the cells | 0 |
| 9 Freedom from degenerative changes in adjacent cartilage |  |
| Normal cellularity. no clusters, normal staining | 3 |
| Normal cellularity, mild clusters, moderate staining | 2 |
| Mild or moderate hypocellularity, slight staining | 1 |
| Severe hypocellularity. poor or no staining | 0 |

This table was adopted from Ref. (Histological assessment of cartilage repair: a report by the Histology Endpoint Committee of the International Cartilage Repair Society (ICRS))
